# Microtubule assembly and pole coalescence: early steps in *Caenorhabditis* *elegans* oocyte meiosis I spindle assembly

**DOI:** 10.1242/bio.052308

**Published:** 2020-06-25

**Authors:** Chien-Hui Chuang, Aleesa J. Schlientz, Jie Yang, Bruce Bowerman

**Affiliations:** Institute of Molecular Biology and Department of Biology, 1229 University of Oregon, Eugene, Oregon 97403, USA

**Keywords:** Meiotic spindle assembly, Microtubules, Oocyte meiosis

## Abstract

How oocytes assemble bipolar meiotic spindles in the absence of centrosomes as microtubule organizing centers remains poorly understood. We have used live cell imaging in *Caenorhabditis elegans* to investigate requirements for the nuclear lamina and for conserved regulators of microtubule dynamics during oocyte meiosis I spindle assembly, assessing these requirements with respect to recently identified spindle assembly steps. We show that the nuclear lamina is required for microtubule bundles to form a peripheral cage-like structure that appears shortly after oocyte nuclear envelope breakdown and surrounds the oocyte chromosomes, although bipolar spindles still assembled in its absence. Although two conserved regulators of microtubule nucleation, RAN-1 and γ-tubulin, are not required for bipolar spindle assembly, both contribute to normal levels of spindle-associated microtubules and spindle assembly dynamics. Finally, the XMAP215 ortholog ZYG-9 and the nearly identical minus-end directed kinesins KLP-15/16 are required for proper assembly of the early cage-like structure of microtubule bundles, and for early spindle pole foci to coalesce into a bipolar structure. Our results provide a framework for assigning molecular mechanisms to recently described steps in *C. elegans* oocyte meiosis I spindle assembly.

## Abstract (precis)

**Summary:** This paper documents requirements for several regulators of microtubule assembly and stability during the early steps of acentrosomal meiotic spindle assembly in *Caenorhabditis elegans* oocytes.

## INTRODUCTION

**Fig. 1.**
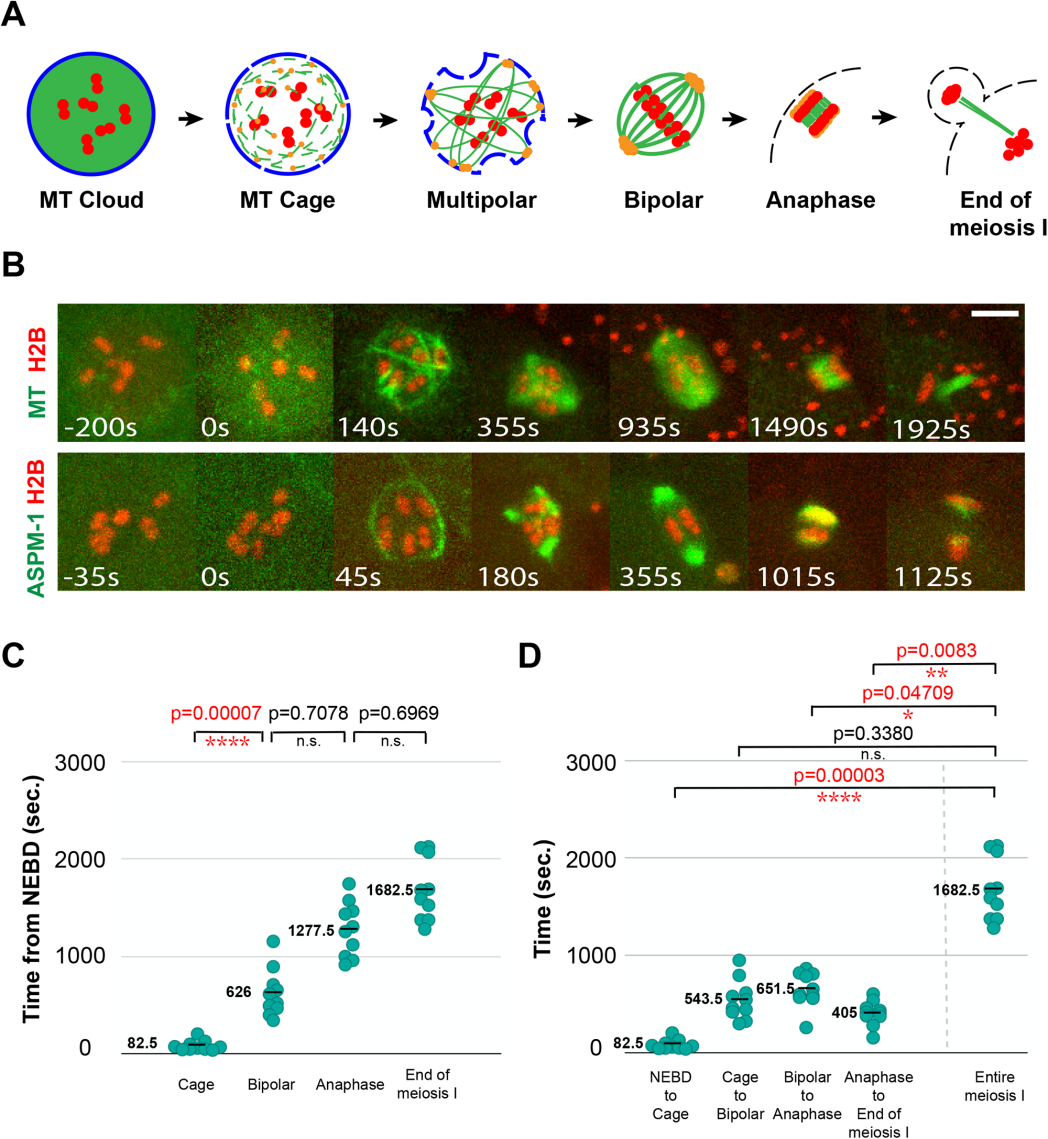
**Wild-type oocyte meiosis I spindle assembly.** (A) Schematic of *C. elegans* oocyte meiosis I spindle assembly. Nuclear lamina (blue), microtubules (MT, green), chromosomes (red), spindle poles (orange) and oocyte plasma membrane (black dashed line) are shown. (B) Time-lapse maximum projection images (see Materials and Methods) of live control oocytes expressing either GFP::TBB-2 and mCherry::H2B to mark microtubules and chromosomes (upper row), or GFP::ASPM-1 and mCherry::H2B to mark spindle poles and chromosomes (lower row). These same control images are used in subsequent figures to provide comparisons to the mutant phenotypes. In this and in all subsequent figures, t=0 is the beginning of NEBD. (C) Scatter plot showing the time from nuclear envelope breakdown (NEBD) to different stages of meiosis I in control oocytes expressing GFP::TBB-2 and mCherry::H2B. The range for each stage was as follows: cage 40–205 s, bipolar 345–1155 s, anaphase 915–1745 s, end of meiosis I 1280–2125 s**.** For all figures, the horizontal bar in the scatter plots and the adjacent number indicate the average value for each dataset, variance was compared using the *F*-test to calculate *P*-values, and the time points for each stage correspond to the earliest time at which each structure was clearly detected. (D) Scatter plot of the times from one stage to the next, and the entire time of meiosis I. The range for each interval was as follows: NEBD to cage 40–205 s, cage to bipolar 300–950 s, bipolar to anaphase 260–865 s, anaphase to end 155–605 s, entire meiosis I 1280–2125 s. Scale bars: 5 μm in all figures.

Oocyte meiotic spindles in many animals form in the absence of centrosomes, and then reduce the duplicated diploid genome to a haploid content through two rounds of cell division called meiosis I and II (Dumont and Desai, 2012; Mullen et al., 2019; Ohkura, 2015; Severson et al., 2016). While acentrosomal microtubule nucleation pathways have been shown to mediate meiotic and mitotic spindle assembly in some settings, how *Caenorhabditis elegans* oocytes nucleate microtubules and assemble bipolar spindles remains poorly understood. However, recent studies have defined a sequence of four steps that assemble bipolar spindles in *C. elegans* oocytes (Gigant et al., 2017; Wolff et al., 2016) (Fig. 1A). First, oocyte nuclear envelope breakdown (NEBD) leads to a diffuse cloud of microtubule signal entering the nucleus. Second, microtubule bundles appear peripherally, just underneath the disassembling nuclear lamina, to form a cage-like structure that surrounds the oocyte chromosomes. Third, the microtubule cage becomes organized such that multiple small foci of microtubule ends form in association with the pole-marker ASPM-1, which initially is present along the entire length of the microtubule bundles that form the early cage-like structure. Finally, these small pole foci coalesce to form a bipolar spindle as chromosomes congress to a metaphase plate (Connolly et al., 2015).

Defining these meiotic spindle assembly steps now provides a foundation for identifying molecular mechanisms that mediate this reductive cell division. While the early microtubule cage assembles in close proximity to the nuclear lamina **(**Wolff et al., 2016**)**, how these microtubules are nucleated and whether the nuclear lamina is required for their assembly are not known. The small GTPase Ran has been implicated in chromosome-mediated nucleation of microtubule assembly during meiosis and mitosis in some organisms ** **(Clarke and Zhang, 2008), but not in *C. elegans* (Askjaer et al., 2002). The augmin complex, which mediates microtubule branching in other animals, is not conserved in *C. elegans* (Edzuka et al., 2014). While microtubule severing by the conserved AAA+ ATPase complex called katanin promotes microtubule density in *C. elegans* oocytes, presumably by generating microtubule fragments that can further elongate (Srayko et al., 2006), mechanism(s) that nucleate microtubule substrates for katanin severing remain unknown. RNAi knockdown of the *C. elegans* γ-tubulin TBG-1 does not prevent oocyte meiotic cell division, but TBG-1 is diffusely associated with oocyte meiotic spindles and its knockdown exacerbates the microtubule loss caused by reduced katanin function (McNally et al., 2006). RNAi-mediated knockdown of two nearly identical minus-end directed *C. elegans* kinesin-14 family members, KLP-15 and -16, destabilizes the cage-like structure (Mullen and Wignall, 2017), consistent with the demonstrated ability of kinesin-14 family members to bundle parallel microtubules (Fink et al., 2009). Finally, the multiple TOG domain protein and XMAP215 family member in *C. elegans*, called ZYG-9, promotes astral microtubule stability during early embryonic mitosis and is known to be required for oocyte meiotic spindle assembly (Bellanger et al., 2007; Bellanger and Gönczy, 2003; Yang et al., 2003). XMAP215 orthologs act as microtubule polymerases in cooperation with γ-tubulin ring complexes (Gunzelmann et al., 2018; Thawani et al., 2018), and have roles in promoting both microtubule stability and instability (Kosco et al., 2001; Shirasu-Hiza et al., 2003), but the role of ZYG-9 in *C. elegans* oocytes remains poorly understood.

To improve our understanding of *C. elegans* oocyte meiosis I spindle assembly, we have used RNAi and mutations to reduce the function of several proteins implicated in this process, and live cell imaging with fluorescent protein fusions to assess their requirements. Here we report our analysis of requirements for the single nuclear lamina protein in *C. elegans* LMN-1 (Liu et al., 2000), the small GTPase RAN-1, the γ-tubulin TBG-1, the kinesin-14 family members KLP-15/16, and the XMAP215 ortholog ZYG-9. Our results indicate that the nuclear lamina is required for assembly of the peripheral cage-like array of KLP-15/16-stabilized microtubule bundles observed early in spindle formation, although bipolar spindles still assembled after LMN-1 knockdown. Furthermore, while not by themselves required for oocyte meiotic cell division, reduction of either RAN-1 or TBG-1 decreased spindle microtubule levels and altered spindle assembly dynamics. Finally, we show that KLP-15/16 and ZYG-9 make distinct contributions both to assembly of the early microtubule cage structure and to the coalescence of pole foci to form a bipolar spindle.

## RESULTS

### Wild-type oocyte meiosis I spindle assembly

To observe early steps in oocyte meiosis I spindle assembly, we imaged control oocytes *in utero* using spinning disk confocal microscopy and simultaneous two-color live imaging (see Materials and Methods), first with a transgenic strain expressing within the germline a GFP fusion to a β-tubulin (GFP::TBB-2) to mark microtubules, and an mCherry fusion to a histone (mCherry::H2B) to mark chromosomes (Fig. 1B; Fig. S1 and Movie 1). To assess progression through meiosis I, we designated the time at which the value for mCherry::H2B intensity in the nucleoplasm became equal to the value for the cytoplasm, which marks the initiation of nuclear envelope breakdown (NEBD), as t=zero and collected z-stacks encompassing the oocyte volume every 5 s. In all ten control oocytes, we observed the previously described steps in spindle assembly: the rapid appearance of GFP::TBB-2 signal around chromosomes, the assembly of peripheral microtubule bundles to form a cage-like structure surrounding the chromosomes, the appearance of a multipolar spindle during prometaphase and the coalescence of multiple small pole foci to form a bipolar spindle by metaphase. Subsequently, during anaphase, most pole microtubules disappeared and central spindle microtubules assembled as the segregating chromosomes moved apart. Finally, we designated the time point with the lowest level of GFP::TBB-2 signal in the spindle area, prior to meiosis II, as the end of meiosis I.

To further characterize spindle assembly, we imaged live control oocytes using a GFP fusion to the pole-marker ASPM-1 (GFP::ASPM-1) and the mCherry::H2B fusion (Fig. 1B; Fig. S2 and Movie 2). As described previously (Gigant et al., 2017; Wolff et al., 2016), GFP::ASPM-1 initially localized along microtubules during cage assembly but then became restricted to multiple small pole foci that coalesced into two poles by metaphase. These GFP::ASPM-1-marked poles subsequently broadened and faded during anaphase.

While all control oocytes in the GFP::TBB-2 background underwent a similar progression in spindle morphology, the time required to complete meiosis I varied from a minimum of 1280 s to a maximum of 2125 s, a 66% increase in duration. To determine when this variability arises, we assessed the variance in timing during progression through the different stages of assembly. The most significant increase in variance occurred during the transition from the appearance of the microtubule cage structure to the establishment of spindle bipolarity (Fig. 1C,D). The subsequent transitions, from the establishment of bipolarity to anaphase onset, and from anaphase onset to the completion of meiosis I, did not show significant further increases. Having collected data sets that established a baseline for wild-type assembly dynamics, we next used RNAi to assess genetic requirements for the early spindle assembly steps and for timely progression through oocyte meiosis I.

We used RNAi to reduce gene function due to the difficulty in defining gene requirements for oocyte meiotic spindle assembly. Essential genes involved in this process often are required for fertile adults to develop, and thus null alleles can result in zygotic embryonic or larval lethality, or adult sterility, precluding their use for definitively analyzing requirements during oocyte meiosis. We therefore have used RNAi to circumvent earlier requirements. We analyzed oocyte meiotic defects at a time point before the RNAi treatment resulted in highly penetrant adult sterility, indicating that their activities were not entirely absent, but after sufficient knockdown resulted in penetrant and previously described defects during early embryonic mitosis (see Materials and Methods). More complete elimination of their functions during oocyte meiosis could result in more severe defects, and the defects we observed could be due to earlier requirements during oogenesis, but RNAi nevertheless allows us to partially reduce gene function and assess progression through oocyte meiotic cell division.

### The nuclear lamina is required for assembly of the peripheral microtubule cage structure

**Fig. 2.**
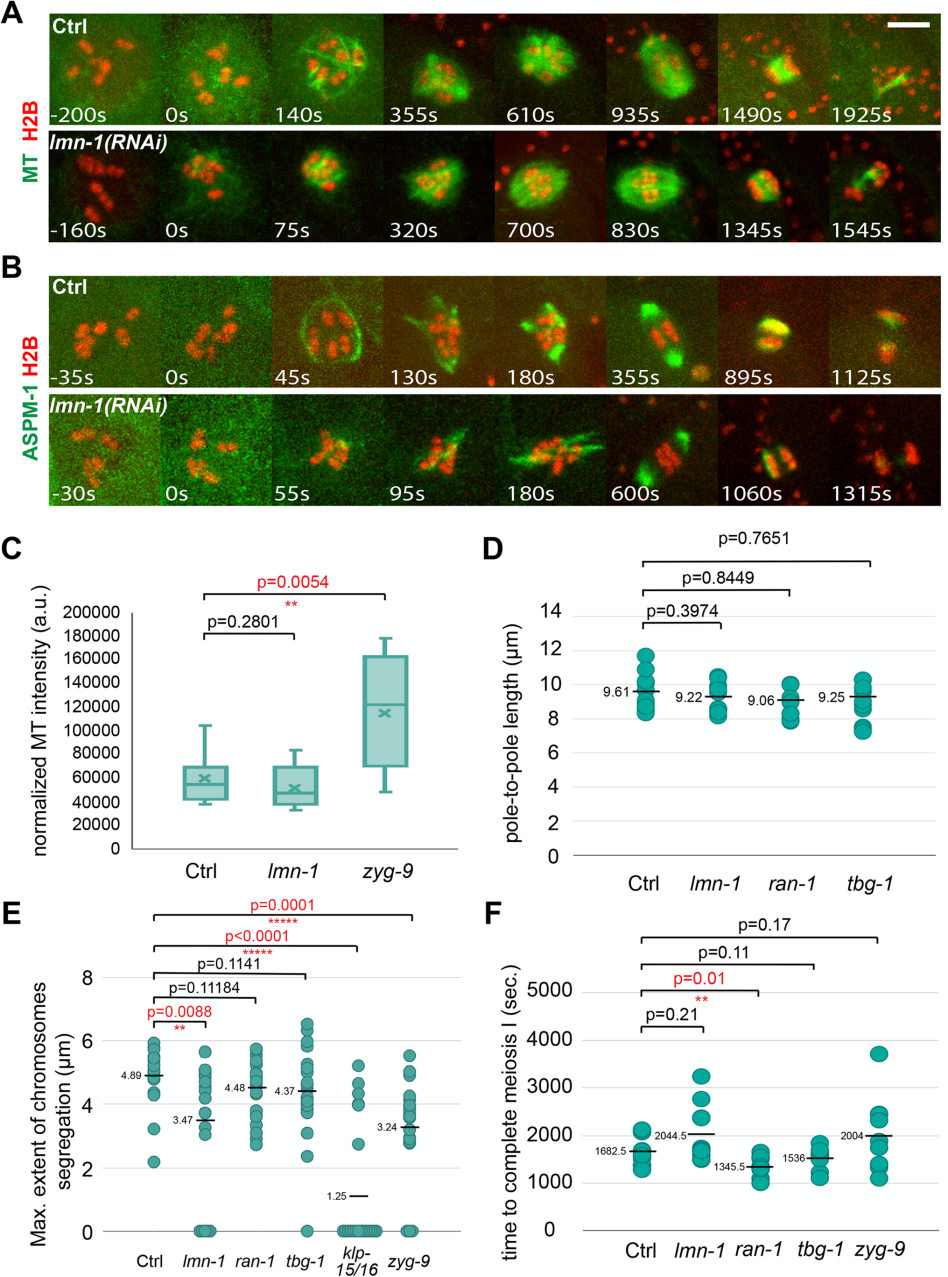
**The nuclear lamina is required for the early cage-like microtubule structure during oocyte meiosis I.** (A,B) Time-lapse maximum projection images during meiosis I of live control and *lmn-1(RNAi)* oocytes expressing either GFP::TBB-2 and mCherry::H2B (A), or GFP::ASPM-1 and mCherry::H2B (B). (C) Normalized microtubule pixel intensity in arbitrary units. The boxplot displays the datasets, with the median (line) and mean (x) values for the three continuous time points with the highest pixel intensities for control and mutant oocytes; bars are 75% and whiskers 95% of the observed normalized microtubule intensity values. (D) Pole-to-pole length measured at metaphase I in control and mutant oocytes expressing GFP::ASPM-1 and mCherry::H2B. For all figures, distributions of scatter plot values were compared using the Mann–Whitney *U*-test to calculate *P*-values. (E) Scatter plot showing maximum extent of chromosome segregation at the end of meiosis I for control and mutant oocytes expressing either GFP::TBB-2 and mCherry::H2B or GFP::ASPM-1 and mCherry::H2B. Numbers of oocytes that failed to segregate chromosomes at the end of meiosis I (i.e. maximum extent of chromosome segregation value=0) were: 5 for *lmn-1(RNAi)*, 1 for *tbg-1(RNAi)*, 12 for *klp-15/16(RNAi)* and 3 for *zyg-9(RNAi)* oocytes. (F) Scatter plot showing the time required to progress through meiosis I in control and mutant oocytes expressing GFP::TBB-2 and mCherry::H2B. Scale bars: 5 μm.

Because the microtubule cage that assembles after the initiation of NEBD is adjacent to and just underneath the nuclear lamina (Wolff et al., 2016), we first asked if the nuclear lamina is required for cage assembly. After using RNAi to knock down the single *C. elegans* lamin LMN-1 in the GFP::TBB-2, mCherry::H2B background, we observed near complete loss of the microtubule cage that forms shortly after NEBD. We did not detect any peripheral microtubule bundles in eight of ten oocytes, and in two oocytes we detected only a few relatively short bundles (Fig. 2A; Fig. S3, Movies 1 and 3). Although the cage was missing or greatly reduced after LMN-1 knockdown, the overall level of microtubule signal was similar to that observed in control oocytes (Fig. 2C) and the subsequent steps in spindle assembly appeared normal, with transient multi-polar structures that coalesced to form bipolar spindles of normal pole-to-pole length (Fig. 2B and D). Moreover, we still observed the disappearance of most microtubules from the poles as central spindle microtubules appeared during anaphase between the segregating chromosome masses, but the extent of chromosome segregation was reduced compared to control oocytes, and in five of 20 oocytes, imaged using either GFP::TBB-2 or GFP::ASPM-1, chromosome segregation completely failed (Fig. 2E). Using the pole marker GFP::ASPM-1, cage-like microtubule bundles were again greatly reduced or absent (Fig. 2B; Fig. S4, Movies 2 and 4). As in control oocytes, GFP::ASPM-1 was initially detected along microtubules as multipolar spindles assembled but never in a structure that resembled the peripheral cage observed in control oocytes. Moreover, the GFP signal persisted along microtubules for a longer period of time before concentrating at pole foci as spindle bipolarity was established. Finally, despite these altered spindle dynamics, the time required to progress through meiosis I, although more variable, was not on average significantly different from control oocytes (Fig. 2F). We conclude that the nuclear lamina is required for assembly of the peripheral microtubule cage that surrounds chromosomes early in meiosis I. While the cage structure was not required for assembly of a bipolar spindle, the dynamics of spindle assembly were altered and chromosome segregation was in some cases entirely absent after LMN-1 knockdown.

### RAN-1 promotes microtubule assembly and normal oocyte nuclear size

We next asked if known regulators of microtubule nucleation might also be required for assembly of the microtubule bundles that form the early cage structure. Previous studies of the *C. elegans* small GTPase RAN-1 and the γ-tubulin family member TBG-1 have not found them to be required for oocyte meiotic cell division (see Introduction). However, the early cage structure is not essential for bipolar spindle assembly after LMN-1 knockdown, and whether cage assembly occurs in the absence of RAN-1 or TBG-1, and the impact of these regulators on spindle assembly dynamics, have not been addressed.

**Fig. 3.**
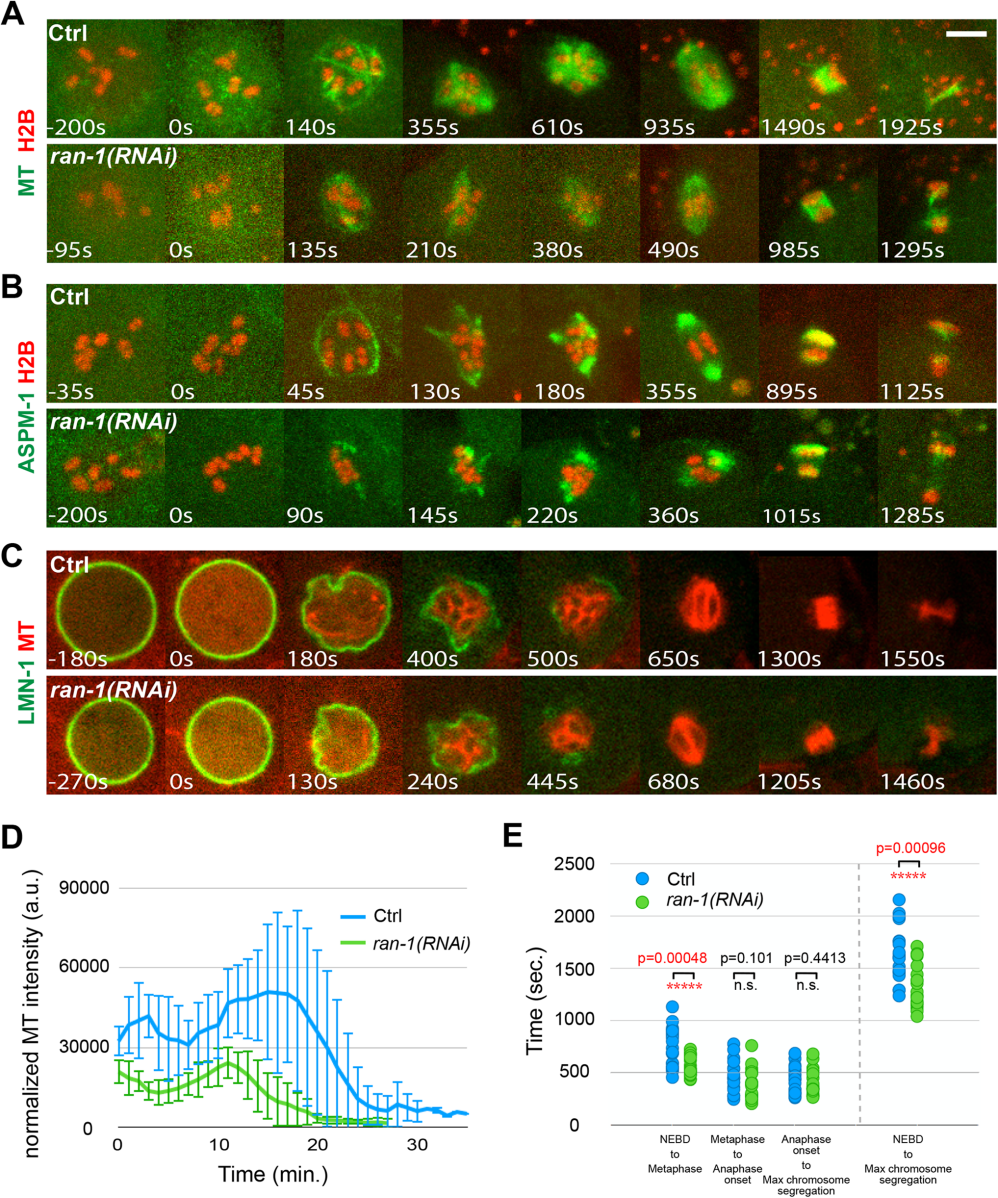
**RAN knockdown reduces oocyte nuclear size and microtubule levels during oocyte meiosis I.** (A–C) Time-lapse maximum projection (A,B) or single focal plane (C) images during meiosis I for live control and *ran-1(RNAi)* oocytes expressing GFP::TBB-2 and mCherry::H2B (A), GFP::ASPM-1 and mCherry::H2B (B), or GFP::LMN-1 and mCherry::TBB-2, to mark the nuclear lamina and microtubules (C). Maximum intensity z-projections (A,B), or the middle plane of the nucleus (C) are shown. (D) Normalized microtubule pixel intensity in arbitrary units measured over time with 1 min time intervals. Time 0=NEBD. For all figures, error bars depict one standard deviation at each time point. (E) Comparison of the length of time from one stage to the next between the control and *ran-1(RNAi)* oocytes from strains expressing either GFP::TBB-2 and mCherry::H2B or GFP::ASPM-1 and mCherry::H2B. NEBD, the time at which the value for histone intensity in the nucleoplasm became equal to the value for the cytoplasm; Metaphase, time at which chromosomes aligned on the metaphase plate; Anaphase onset, the time at which chromosomes started to separate; Maximum chromosome segregation, the time at which chromosomes separated to the maximum distance. Scale bars: 5 μm.

We used RNAi to knock down RAN-1 in strains expressing GFP::TBB-2 and mCherry::H2B (Fig. 3A and Fig. S6), GFP::ASPM-1 and mCherry::H2B (Fig. 3B and Fig. S7), and GFP::LMN-1 and mCherry::TBB-2 (Fig. 3C). We observed a roughly normal sequence of spindle morphology transitions, with a microtubule and ASPM-1 cage forming soon after NEBD, followed by the appearance of multiple small pole foci that coalesced to form a bipolar spindle of normal length that segregated chromosomes to a normal extent (Fig. 2D,E, Fig. 3A,B; Figs S6 and S7). However, compared to control oocytes, the quantities of microtubules detected throughout meiosis I were reduced (Fig. 3D), and the microtubule cage was smaller in diameter (Fig. 3A,B;Figs S6, S7 and S8A). Furthermore, the time required to complete meiosis I, based on GFP::TBB-2 imaging (Fig. 2F), was reduced from an average of 1682.5 s in control oocytes to an average of 1345.5 s after RAN-1 knockdown (*P*=0.01). We further assessed progression through meiosis I based on chromosome dynamics and observed a significant decrease during the time from NEBD to metaphase (Fig. 3E). We also examined spindle pole dynamics with GFP::ASPM-1 and again found that the decrease in time occurred during the establishment of spindle bipolarity, between NEBD and metaphase (Fig. S5A). To summarize, RAN-1 is at least partially required for spindle microtubule assembly during meiosis I but may not be required for a functional bipolar spindle to form. Surprisingly, reducing RAN-1 function leads to a more rapid progression through meiosis I that may result from a reduction in the time required for early pole foci to coalesce and form a bipolar spindle.

We next asked if the smaller microtubule cage observed after RAN-1 knockdown is associated with a smaller oocyte nucleus, or if the cage forms more internally relative to the nuclear lamina in normally sized nuclei, by knocking down RAN-1 in a transgenic strain expressing GFP::LMN-1 and an mCherry fusion to TBB-2 (Fig. 3C). While the timing of NEBD based on the fragmentation and decrease of the GFP::LMN-1 signal over time appeared normal, the initial diameter of the nuclear lamina structure was reduced to roughly the same extent as for the microtubule cage (Fig. 3A–C;Fig. S8A and B). Similarly, the diameters of oocyte nuclei measured using Nomarski optics were reduced relative to control oocytes (Fig. S8C and D). Consistent with these findings, RAN-1 knockdown has previously been reported to result in abnormally small oocyte pronuclei (Askjaer et al., 2002).

One possible explanation for the more rapid progression through meiosis I after RAN-1 knockdown is that the smaller size of the oocyte nucleus results in chromosomes occupying a smaller volume after NEBD, leading to more rapid spindle assembly and chromosome capture. We therefore measured the area occupied by chromosomes (AOC) from NEBD to metaphase but did not observe a significant decrease after RAN-1 knockdown compared to control oocytes (Fig. S8E and F). We also measured the AOC from NEBD to metaphase after LMN-1 knockdown, which as noted above failed to assemble a peripheral cage structure, and observed a substantial decrease in this area compared to control oocytes (Fig. S8E), even though the diameter of oocyte nuclei were no different from control oocytes (Fig. S8C and D). Nevertheless, the time required to progress through meiosis I after LMN-1 knockdown was not significantly reduced compared to control oocytes (Fig. 2F). We therefore suspect that RAN-1 knockdown oocytes progressed through meiosis I more rapidly due to the reduced diameter of the cage structure, and not to a decrease in the AOC (see Discussion).

### TBG-1 is required for proper oocyte nuclear positioning and promotes both microtubule levels and normal spindle assembly dynamics

**Fig. 4.**
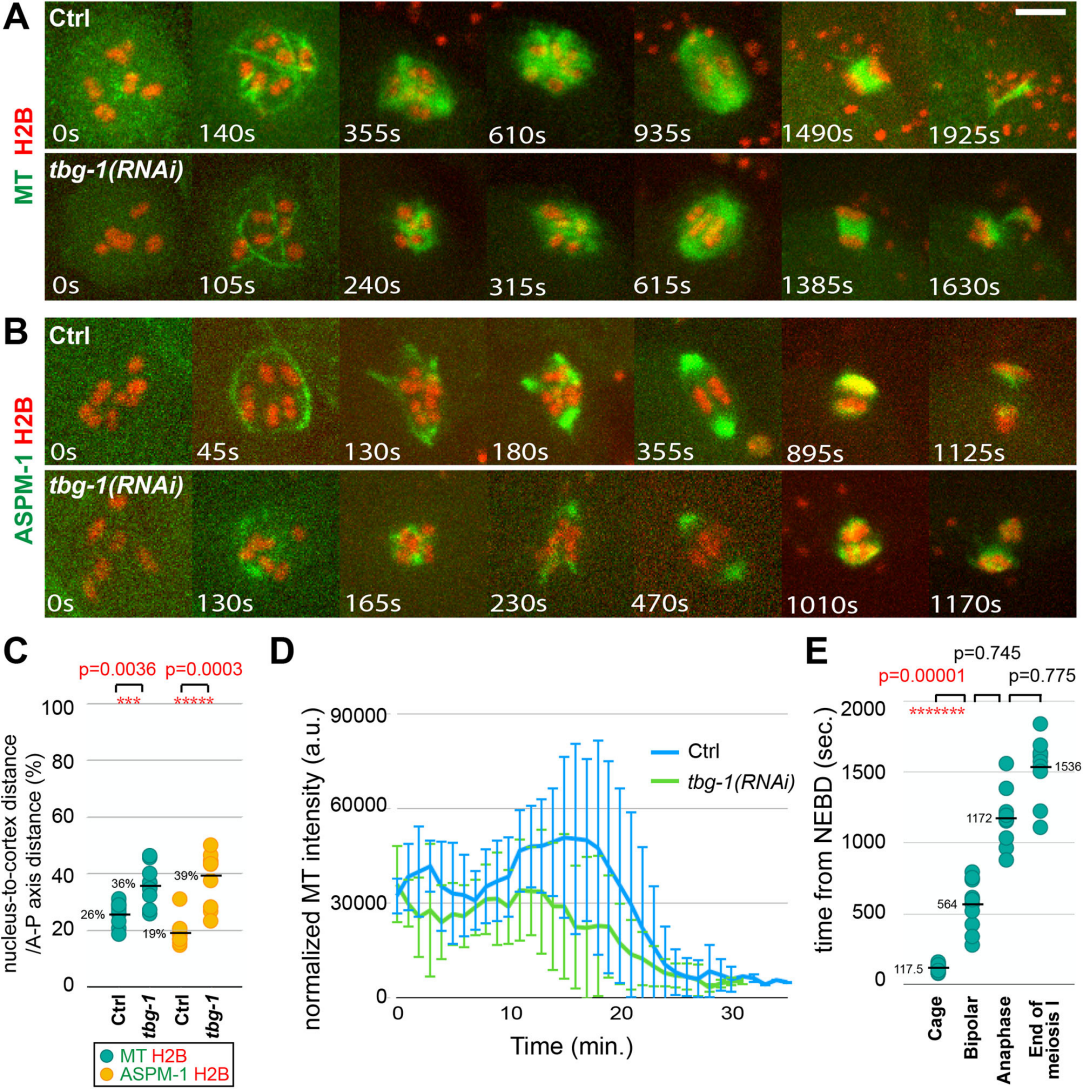
**TBG-1 knockdown alters spindle dynamics after the microtubule cage-like structure appears and reduces microtubule levels during oocyte meiosis I.** (A,B) Time-lapse maximum projection images during meiosis I of live control and *tbg-1(RNAi)* oocytes expressing either GFP::TBB-2 and mCherry::H2B (A), or GFP::ASPM-1 and mCherry::H2B (B). (C) Scatter plot showing nuclear position for control and *tbg-1(RNAi)* oocytes, measured at NEBD as the distance from the center of the nucleus to the closest edge of the oocyte on the same focal plane/the length of oocyte anterior-posterior axis. (D) Normalized microtubule pixel intensity in arbitrary units measured over time with 1 min time intervals. Time 0=NEBD. (E) Scatter plot showing the time from NEBD to different stages of meiosis I in *tbg-1(RNAi)* oocytes expressing GFP::TBB-2 and mCherry::H2B. The mean values for control oocytes are: cage 82.5 s, bipolar 626 s, anaphase 1277.5 s, end of meiosis I 1682.5 s. Scale bars: 5 μm.

We next examined spindle assembly after knocking down TBG-1 in transgenic strains expressing GFP::TBB-2 and mCherry::H2B (Fig. 4A; Fig. S9), or GFP::ASPM-1 and mCherry::H2B (Fig. 4B; Fig. S10). Prior to NEBD and spindle assembly, we observed a displacement of oocyte nuclei from the cortex (Fig. 4C), followed by a reduction in microtubule levels throughout meiosis I (Fig. 4D). The diameter of the microtubule cage structure, though more variable, was on average not significantly different from its diameter in control oocytes (Fig. S8A), but the subsequent dynamics of spindle assembly were distinct from those observed in both control and RAN-1 knockdown oocytes. Shortly after cage assembly, the microtubules collapsed into a small cluster around the oocyte chromosomes, rather than forming the peripheral multipolar structure observed in control oocytes. Similarly, GFP::ASPM-1 foci and the oocyte chromosomes became grouped together in a tight cluster. Subsequently, more extended microtubule structures appeared and multiple GFP::ASPM-1 foci emerged peripherally to the oocyte chromosomes and coalesced to form a bipolar spindle of normal length that segregated chromosomes to a normal though more variable extent (Fig. 2D,E). In one case, when imaging GFP::ASPM-1, we observed a complete failure in chromosome segregation (Fig. 2E). Finally, the time to complete meiosis I and the variance in the time required to achieve spindle bipolarity were similar to control oocytes (Figs 1C, 2F and 4E). In summary, as reported previously (McNally et al., 2006), TBG-1 was not required for bipolar spindle assembly. However, microtubule levels were reduced and the dynamics of spindle assembly were altered and distinct from those observed after RAN-1 knockdown*.*

### KLP-15 and -16 are required for cage stability and promote pole coalescence

The nearly identical *C. elegans* minus-end directed kinesin-14 family members KLP-15 and -16 have been shown previously to have a role in stabilizing the microtubule bundles that form the early cage structure (Mullen and Wignall, 2017). After RNAi knockdown of KLP-15/16, the bundles were less prominent compared to control oocytes and a more diffuse spherical distribution of microtubules then surrounded the chromosomes, which underwent segregation in only about one third of the depleted oocytes. As these results were obtained using RNAi, and spindle structures were assessed using immunofluorescence in fixed oocytes, we have analyzed KLP-15/16 requirements using live cell imaging with putative null alleles.

**Fig. 5.**
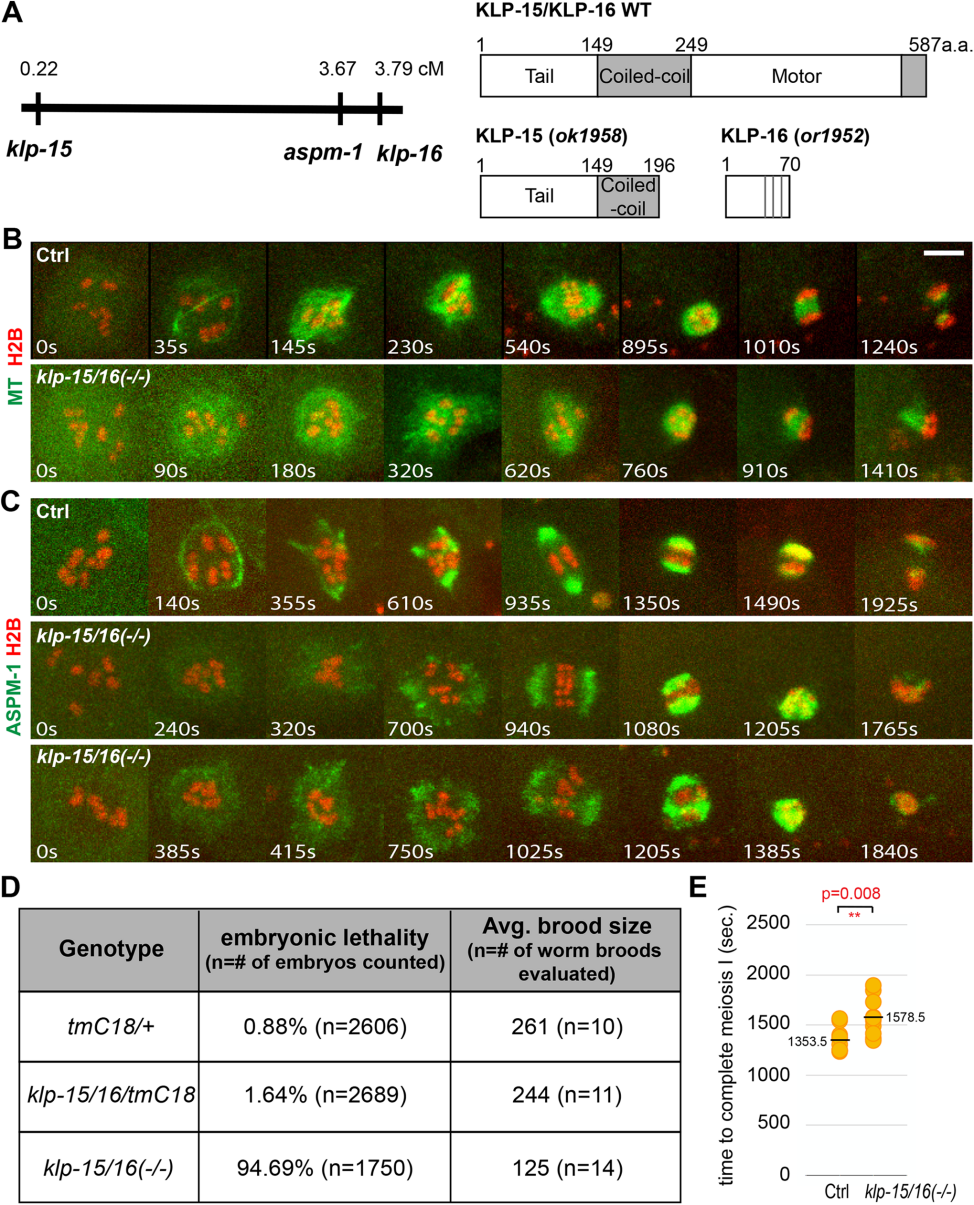
**KLP-15/16 knockdown destabilizes cage-like microtubule bundles and reduces pole coalescence.** (A) Left panel: locations of *klp-15*, *aspm-1* and *klp-16* on chromosome I. Right panel: domain architectures of wild-type KLP-15/16 and the KLP-15/16 double mutant used in this study. Vertical grey lines indicate stop codons that follow the frameshift caused by the *or1952* deletion. (B) Time-lapse maximum projection images during meiosis I of control and *klp-15/16* double mutant oocytes expressing GFP::TBB-2 and mCherry::H2B. (C) Time-lapse maximum projection images during meiosis 1 of control or *klp-15*/*16* double-mutant oocytes expressing GFP::ASPM-1 and mCherry::H2B. (D) Embryonic lethality and average brood sizes for *klp-15*/*16* double-mutant and control strains. (E) Scatter plot showing the time required to progress through meiosis I for control and *klp-15*/*16* double-mutant oocytes. Scale bars: 5 μm.

We first made a strain carrying likely null alleles for both *klp-15* and *-16*, which are on the same chromosome separated by about 3.5 map units (Fig. 5A). Using CRISPR/Cas9, we introduced a small deletion near the 5′ end of the first coding exon of *klp-16* in a strain homozygous for a previously isolated and homozygous viable *klp-15* deletion allele, *klp-15(ok1958)*. The CRISPR-generated *klp-16* deletion resulted in a frameshift followed by multiple stop codons and likely eliminates all gene function. The resultant double-mutant chromosome, *klp-15(ok1958) klp-16(or1952)* was then balanced with a marked inversion chromosome, *tmC18* (Dejima et al., 2018). When homozygous, these two mutations resulted in the development of fertile adults with reduced brood sizes and recessive and penetrant embryonic lethality (Fig. 5D).

We next used live imaging to examine oocyte meiosis I spindle assembly in the *klp-15/16* null mutant background. We used genetic crosses to generate a *tmC18* balanced *klp-15/16* double mutant strain that expresses GFP::TBB-2 and mCherry::H2B (Fig. 5B; Fig. S11). Because the *aspm-1* gene resides on the same chromosome between *klp-15* and *-16* (Fig. 5A), we obtained a CRISPR/Cas9-generated *in situ* GFP fusion to the endogenous *aspm-1* locus on the *klp-15(or1958) klp-16(or1952)* chromosome (see Materials and Methods) and used genetic crosses to introduce the mCherry::H2B fusion (Fig. 5C; Fig. S12). As reported previously (Mullen and Wignall, 2017), we observed with both GFP::TBB-2 and GFP::ASPM-1 a decreased prominence of the microtubule bundles that form the cage (Fig. 5B and C, Figs S11 and S12). Subsequently, the microtubules formed a ball-like structure that failed to establish obvious bipolarity (Fig. 5B; Fig. S11) and chromosome segregation failed in 60% of the mutant oocytes (Fig. 2E; Figs S11 and S12). While the microtubules did not form obviously bipolar spindles in the mutant oocytes, we observed the assembly of abnormal bipolar spindle structures with GFP::ASPM-1 live imaging (Fig. 5C; Fig. S12, Movies 2 and 5). GFP::ASPM-1 foci initially were detected peripheral to the chromosomes, became more prominent over time, and often formed broad poles that failed to coalesce into the more compact structures observed in control oocytes (Fig. 1B, Fig. 5C; Fig. S2, Fig. S12 and Movies 6–8). In some cases, broad arrays of GFP::ASPM-1 foci nearly encircled the chromosomes and the average time to complete meiosis I was increased (Fig. 5E). We conclude that in addition to being important for stable assembly of the microtubule cage and chromosome segregation, KLP-15 and -16 are important for the coalescence of early pole foci into properly organized spindle poles.

### ZYG-9 restricts microtubule bundles to the periphery during cage assembly and promotes pole coalescence and stability

**Fig. 6.**
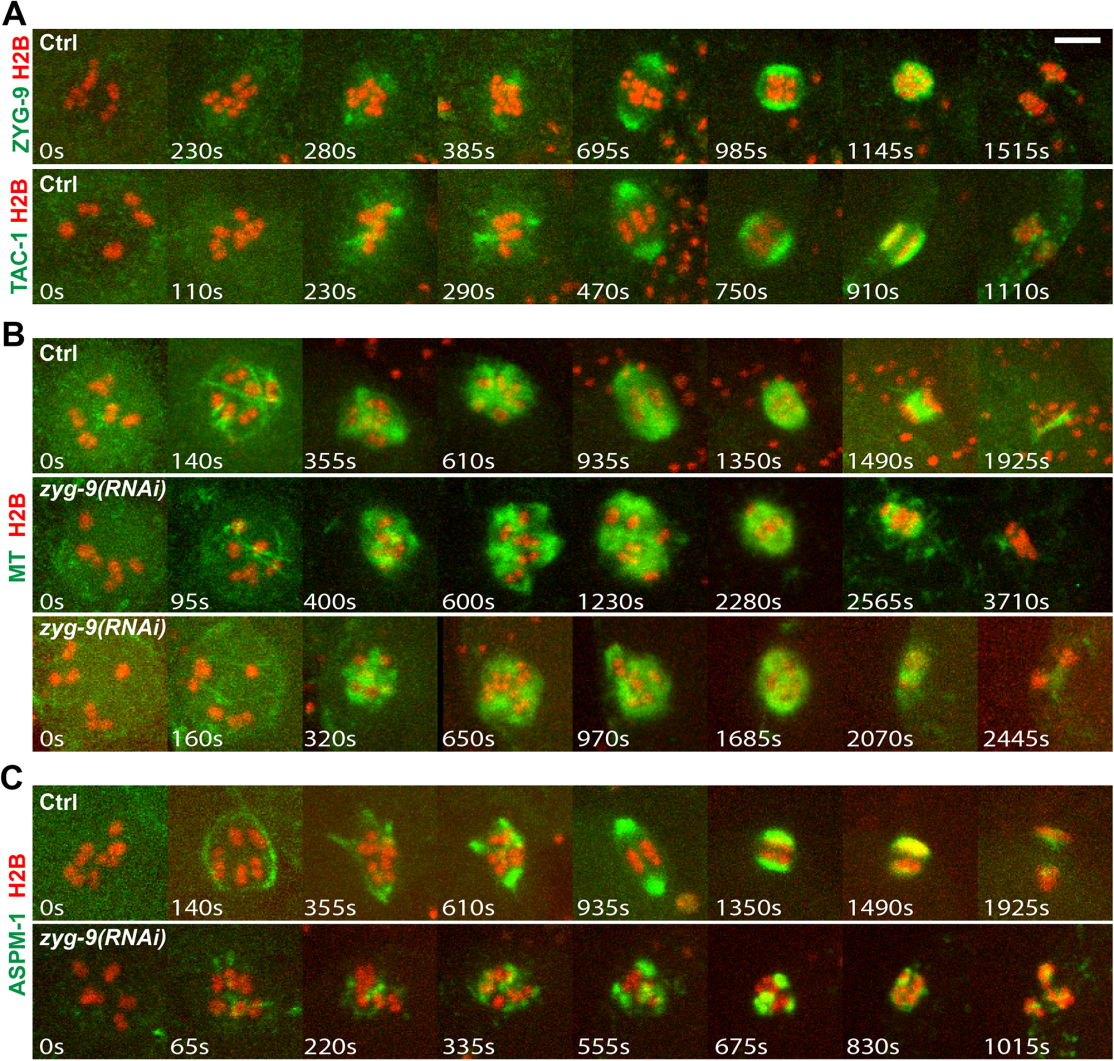
**ZYG-9 knockdown causes spindle assembly defects throughout oocyte meiosis I.** (A) Time-lapse maximum projection images during meiosis I of live control oocytes expressing either GFP::ZYG-9 and mCherry::H2B (upper row), or GFP::TAC-1 and mCherry::H2B (lower row). (B,C) Time-lapse maximum projection images during meiosis I of live control and *zyg-9(RNAi)* oocytes expressing either GFP::TBB-2 and mCherry::H2B (B), or GFP::ASPM-1 and mCherry::H2B (C). Scale bars: 5 μm.

The XMAP215 family member ZYG-9 is known to be required for oocyte meiotic spindle assembly (Yang et al., 2003), but the nature of this requirement remains poorly understood. To examine its role, we first assessed the dynamics of ZYG-9 localization in comparison to microtubules and ASPM-1. We used CRISPR/Cas9 to generate an *in situ* fusion of GFP to the endogenous *zyg-9* locus and genetic crosses to introduce the mCherry::H2B fusion (Fig. 6A and Fig. S15A). In contrast to GFP::ASPM-1, we did not detect GFP::ZYG-9 in association with the microtubule cage; rather, it was initially more diffusely present near chromosomes, subsequently became enriched at multiple pole foci and also was present more diffusely throughout the spindle during pole coalescence. Upon the establishment of spindle bipolarity, GFP::ZYG-9 was enriched at the poles but in contrast to GFP::ASPM-1, GFP::ZYG-9 also was detected between the poles. ZYG-9 binds the coiled-coil TACC ortholog TAC-1, and both promote microtubule stability during early embryonic mitosis in *C. elegans* (Bellanger et al., 2007; Bellanger and Gönczy, 2003). We therefore used CRISPR/Cas9 to generate an *in situ* fusion of GFP to the endogenous *tac-1* locus and observed localization dynamics similar to GFP::ZYG-9 (Fig. 6A;Fig. S15B). To summarize, ZYG-9 and its partner TAC-1 were both present throughout spindle assembly and both appeared more restricted in distribution than microtubules but, as assembly progressed, more broadly distributed than ASPM-1.

We next examined ZYG-9 requirements after RNAi knockdown in transgenic strains expressing GFP or mCherry fusions to TBB-2, ASPM-1 and H2B and observed multiple defects during meiosis I spindle assembly (Fig. 6B and C; Figs S13 and S14). First, when imaging spindles marked with GFP::TBB-2 or GFP::ASPM-1, microtubule bundles assembled to form a peripheral cage shortly after NEBD, but some of the microtubule bundles were not restricted to the periphery and instead passed through the interior of the chromosome occupied space, which we detected using Imaris to generate and rotate 3-D reconstructions (Movies 9–14). Subsequently, foci of GFP::TBB-2 and GFP::ASPM-1 were observed not only at the periphery surrounding oocyte chromosomes, but also between some of the bivalent chromosomes. Moreover, small spindle-like structures often appeared to form around individual or small groups of bivalents, in contrast to the peripheral spindle foci that coalesced to form a bipolar spindle in control oocytes.

**Fig. 7.**
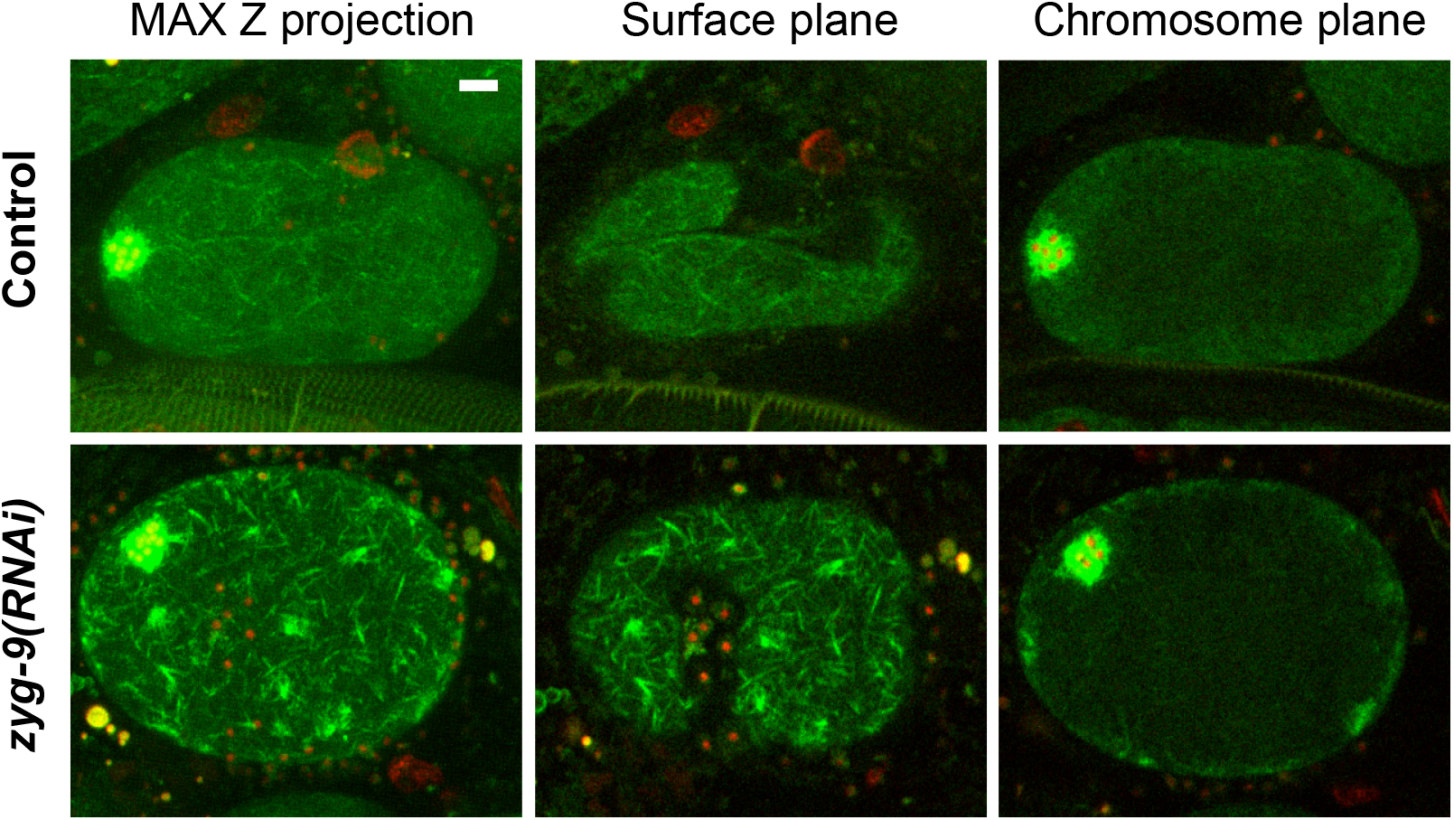
**Microtubule levels are increased throughout the oocyte cortex during meiosis I after ZYG-9 knockdown.** *Ex utero* spinning disk confocal images for live control (upper row) and *zyg-9(RNAi)* (lower row) oocytes expressing GFP::TBB-2 and mCherry::H2B. Pixel intensity was enhanced to in the green channel to highlight cortical microtubules. Left panels: maximum intensity z-projection image of 15 planes with 1 μm z-spacing. Middle panels: surface plane. Right panels: chromosome plane. Scale bars: 5 μm.

The abnormal dynamics of spindle assembly observed after ZYG-9 knockdown were accompanied by a significant increase in the level of oocyte spindle microtubules (Figs 2C and 6B; Fig. S13; Movies 15 and 16), and a variable but significant increase in the diameter of the microtubule cage structure (Fig. S8A). We also observed increased microtubule levels throughout the oocyte cortex during meiosis I (Fig. 7). These increases were surprising because ZYG-9 is required for the stability of astral microtubules during early embryonic mitosis (Bellanger et al., 2007; Bellanger and Gönczy, 2003), and ZYG-9 orthologs in other species promote microtubule assembly (Akhmanova and Steinmetz, 2015), although in some contexts they also promote microtubule instability (Shirasu-Hiza et al., 2003).

We also observed a striking lack of pole stability and extensive chromosome segregation defects after ZYG-9 knockdown. In control oocytes, early small pole foci stably associated with each other over time (Fig. 1B; Figs S2 and S16, Movies 2 and 17). In contrast, after ZYG-9 knockdown, pole foci marked by GFP::ASPM-1 fused and then often broke apart as meiosis I progressed (Fig. 6C; Fig. S16 and Movies 18–20). Consistent with a role in pole stability, chromosomes sometimes segregated into three masses during anaphase (ten of 20 oocytes), although in other cases no segregation (three of 20 oocytes) or segregation into two masses (seven of 20 oocytes) were observed (Figs S5C, S13 and S14). Finally, we observed similar defects throughout oocyte meiosis I after knocking down the TAC-1 binding partner for ZYG-9 (Movies 21 and 22), indicating that ZYG-9 and TAC-1 have similar if not identical requirements. In summary, ZYG-9 and TAC-1 restrict cage microtubule bundles to the periphery and promote pole coalescence and stability. Notably, they also appear to limit both spindle and cortical microtubule levels during oocyte meiosis I.

## DISCUSSION

We have examined the requirements for several factors involved in *C. elegans* oocyte meiotic spindle assembly, with the goal of assessing their roles during a sequence of four recently described assembly steps that generate these acentrosomal and yet bipolar spindles during meiosis I. Our results show that the nuclear lamina is required for assembly of the peripheral microtubule cage, although this structure is not required for bipolar spindle assembly. While knockdown of either of two conserved regulators of microtubule nucleation, the small GTPase RAN-1 and TBG-1/γ-tubulin, did not prevent bipolar spindle assembly or chromosome segregation, microtubule levels after both knockdowns were reduced and spindle assembly dynamics were altered. We also identified two additional contributors to assembly of the early cage-like network of microtubule bundles. After knockdown of the XMAP215 ortholog ZYG-9, or its binding partner TAC-1, microtubule bundles were no longer restricted to the periphery but in some cases passed through the space occupied by oocyte chromosomes. As reported previously, these microtubule bundles were reduced in prominence in mutants lacking the nearly identical minus-end directed kinesins KLP-15 and -16. Finally, both ZYG-9 and KLP-15/16 were required for early spindle pole foci to coalesce into a bipolar structure, but in distinct ways. ZYG-9 knockdown resulted in a lack of pole stability during coalescence, while pole foci failed to coalesce in *klp-15/16* mutant oocytes. Our results, considered in more detail below, document requirements for microtubule nucleation, cage assembly and pole coalescence, key steps in the assembly of *C. elegans* acentrosomal oocyte meiosis I spindles.

### Microtubule nucleation and *C. elegans* oocyte meiotic spindle assembly

Two widely conserved regulators that contribute to microtubule nucleation in other contexts, γ-tubulin and the small GTPase Ran, have not been found to be required for oocyte meiotic cell division in *C. elegans* (see Introduction). However, our results indicate that both contribute to producing normal microtubule levels and spindle assembly dynamics. RAN-1 knockdown did not substantially affect any of the steps in oocyte spindle assembly but did result in the production of oocyte nuclei and a microtubule cage that were reduced in diameter. Moreover, microtubule levels were reduced and declined to a minimum earlier than was observed in control oocytes, and the time required to progress through meiosis I was reduced. TBG-1 knockdown also did not prevent assembly of the microtubule cage, and its diameter was similar to those in control oocytes. However, microtubule levels were reduced and the cage quickly collapsed into a condensed ball of microtubule signal surrounding the chromosomes. Subsequently, microtubules emerged from the collapsed structure, and the pole marker ASPM-1 appeared in multiple foci that coalesced to form a bipolar spindle of normal length that segregated chromosomes to the same extent as in control oocytes. In spite of these changes in spindle assembly dynamics, we did not detect any defects in chromosome segregation by the end of meiosis I after knockdown of either RAN-1 or TBG-1, with one exception after TBG-1 knockdown, consistent with previous reports indicating the lack of an essential requirement for either of these regulators during oocyte meiosis.

While the more normal sequence of assembly events after RAN-1 knockdown and the more substantially altered assembly dynamics after TBG-1 knockdown suggest that these two regulators play distinct roles, in neither case have we been able to determine the consequences of fully eliminating gene function. For both RAN-1 and TBG-1, we analyzed oocyte meiotic spindle defects at a time prior to which the RNAi treatments resulted in adult sterility (see Materials and Methods), indicating that their activities were not entirely absent. More complete elimination of their functions could result in more severe and perhaps more similar defects. We attempted to knock down both genes simultaneously, using both feeding and microinjection RNAi, but did not observe more severe phenotypes (data not shown). Because the time course of feeding RNAi required to achieve maximal knockdown while retaining fertility was different for each gene, and because the use of RNAi to knock down multiple genes simultaneously can be less effective than single gene RNAi knockdowns, these negative results may simply reflect an incomplete loss of gene function. An alternative approach to reducing gene function at different times in development is to use CRISPR/Cas9 to tag endogenous loci with a degron motif that induces degradation of the tagged protein upon treatment with the plant hormone auxin (Zhang et al., 2015). While this approach does not allow one to determine null phenotypes conclusively, it can be especially useful for simultaneously reducing the functions of multiple degron-tagged proteins. Future experiments using degron tagging may facilitate better assessment of the potentially distinct roles of RAN-1 and TBG-1.

### The nuclear lamina as a platform for assembling the cage-like network of microtubule bundles

While we did not detect a requirement for either RAN-1 or TBG-1 in assembly of the early microtubule cage, we did find that the nuclear lamina, which directly overlies this structure, is required for its assembly. RNAi knockdown of the only *C. elegans* lamin LMN-1 nearly eliminated the peripheral microtubule bundles. We also observed other defects in spindle assembly dynamics after LMN-1 knockdown. The pole marker ASPM-1 persisted along the length of microtubules for a longer period of time before becoming enriched at the two spindle poles compared to control oocytes, but bipolar spindles of normal length ultimately assembled and the time required to complete meiosis I was more variable but not significantly increased. While alternative bipolar spindle assembly mechanisms appear to compensate for loss of the microtubule cage structure, chromosomes were segregated to a lesser extent, and in five of 20 cases chromosome segregation failed.

Restricting the assembly of early microtubule bundles to the periphery to form the cage might promote pole coalescence by having it occur only along the inner surface of the nuclear lamina, rather than throughout the volume occupied by oocyte chromosomes. Consistent with such a role, the time required to complete meiosis I in control oocytes differs due to variability in the time required for pole coalescence, and the reduced time required to complete meiosis after RAN-1 knockdown occurs during pole coalescence and correlates with a reduction in the diameter and hence the surface area of the cage-like network. While the absence of a cage after LMN-1 knockdown did not increase the time required to complete meiosis I, it did result in defects in chromosome segregation. We therefore suggest that the cage may be important for assembly of a functional bipolar spindle in the absence of centrosomes, with the diameter of the cage influencing the time required for pole coalescence.

Some or all of the defects we observed after LMN-1 knockdown could be indirectly due to disruptions in the nuclear import of factors required for a fully functional spindle to assemble. While we did not detect obvious differences after RAN-1 or LMN-1 knockdown in the dynamics of mCherry::H2B and GFP::TBB-2 distribution before and after NEBD, compared to control oocytes (Figs 1A, 2A,B, 3A and C), we did detect variable and sometimes lower levels of some kinetochore proteins after LMN-1 knockdown (data not shown). While our results do not distinguish between indirect and direct roles, we did observe distinct defects after knockdown of LMN-1 or RAN-1. Thus the different outcomes might reflect distinct requirements for these two factors, in either nuclear import/export or spindle assembly. In either case, our results indicate that the nuclear lamina plays an important role in *C. elegans* oocyte meiotic spindle assembly, and roles for lamins in spindle assembly have also been reported in *Xenopus* extracts and during *Drosophila* male meiosis (Goodman et al., 2010; Hayashi et al., 2016; Tsai et al., 2006).

### ZYG-9/XMAP215 and the kinesin-14 family members KLP-15/16 contribute to proper assembly of the cage-like network of microtubule bundles

We also found requirements for ZYG-9 and the minus-end directed kinesins KLP-15/16 in the assembly of the microtubule cage structure. As reported previously based on RNAi knockdown (Mullen and Wignall, 2017), we found that the cage microtubule bundles that formed in oocytes from worms homozygous for likely null alleles of *klp-15* and *-16* were less prominent and rapidly became undetectable, with the microtubules instead forming a diffuse cloud encompassing the oocyte chromosomes, and chromosome segregation often completely failed. While Mullen and Wignall (2017) reported that chromosome segregation failed in only about one third of *klp-15*/*16* mutant oocytes after RNAi knockdown, the more highly penetrant chromosome segregation defects we observed (60%) are likely due to our use of putative null alleles.

We observed a very different defect in the microtubule cage structure after depletion of ZYG-9/XMAP215, or of its binding partner TAC-1. Stable and prominent microtubule bundles formed peripherally to the oocyte chromosomes early in spindle assembly, as in control oocytes, but some bundles passed through the interior of the chromosome occupied volume. Subsequently, some pole foci formed among the chromosomes, and oocytes often failed to assemble bipolar spindles. In some oocytes, the spindles became tripolar and segregated chromosomes into three distinct masses, while in other cases chromosome segregation completely failed. We conclude that ZYG-9 and KLP-15/16 make distinct contributions to assembly of the cage-like network of microtubule bundles: KLP-15/16 are required for their stability, while ZYG-9 restricts their assembly to the periphery.

We also observed increased levels both of spindle-associated microtubules and of microtubules throughout the oocyte cortex during meiosis I after ZYG-9 knockdown. This was surprising given that ZYG-9 orthologs have been reported to promote microtubule stability and act as microtubule polymerases (Akhmanova and Steinmetz, 2015). Indeed, astral microtubules during early embryonic mitosis in *C. elegans zyg-9* mutants are abnormally short (Bellanger et al., 2007; Bellanger and Gönczy, 2003). Nevertheless, other studies also have reported a role for XMAP215 orthologs in promoting instability (Brittle and Ohkura, 2005; Shirasu-Hiza et al., 2003), and our analysis of ZYG-9 provides further evidence that these TOG domain proteins can promote microtubule instability. Moreover, ZYG-9 can promote both microtubule stability and microtubule instability depending on the cellular context, even when the different activities are closely spaced in time, as also appears to be true for the budding yeast ortholog (Kosco et al., 2001; Shirasu-Hiza et al., 2003). Finally, our results indicate that ZYG-9 acts in concert with its conserved binding partner, TAC-1, not only to promote microtubule stability during early embryonic mitosis, but also to promote microtubule instability during oocyte meiosis I.

A role for ZYG-9 in promoting microtubule instability may account for the spatial organization of the microtubule bundles that surround the oocyte chromosomes early in meiosis I spindle assembly. GFP::ZYG-9 and GFP::TAC-1 initially were distributed diffusely throughout the space occupied by chromosomes and were not detected in association with the peripheral microtubule bundles. Thus, ZYG-9 and TAC-1 might prevent microtubule assembly in the volume occupied by chromosomes, restricting cage formation to the periphery.

### ZYG-9/XMAP215 and the kinesin-14 family members KLP-15/16 make distinct contributions to pole coalescence

ZYG-9 and KLP-15/16 also make distinct contributions to pole coalescence. In control oocytes, multiple small GFP::ASPM-1 pole foci moved toward each other and fused upon coming into contact, only rarely undergoing fission into distinct foci after merging. How these foci move toward each other remains unknown, but a similar process of coalescence has been observed in mouse oocytes (Schuh and Ellenberg, 2007). After ZYG-9 knockdown, we observed frequent examples of pole instability, in which pole foci would merge but then split apart. This process continued for an extended period with spindles frequently failing to become bipolar and often segregating chromosomes into three masses instead of two, or entirely failing to segregate chromosomes. By contrast, in *klp-15*/*16* mutant oocytes, pole foci became more prominent over time but were less dynamic. Although these foci often formed bipolar structures, the poles were much broader, and in some cases they nearly encircled the chromosomes without ever forming distinct poles. In addition, chromosome segregation often failed entirely. In contrast to our results using live imaging with an endogenous fusion of GFP to ASPM-1, Mullen and Wignall (2017) concluded that *klp-15*/*16* mutant oocytes fail to assemble bipolar spindles, based on their analysis of fixed oocytes. Our results indicate that while pole coalescence is highly defective in *klp-15*/*16*-null mutants, some spindle bipolarity nevertheless emerges but usually is insufficient to promote chromosome segregation. We conclude that while KLP-15/16 promote pole coalescence, ZYG-9 promotes pole stability, with both playing important roles in establishing a bipolar spindle.

Our results do not provide direct mechanistic insight into how either ZYG-9 or KLP-15/16 promote pole stability and coalescence, but the known functions of these conserved proteins may be relevant. Mutations in the *Drosophila* ortholog of KLP-15/16, Ncd, also result in disorganized oocyte meiotic spindles, with unfocused poles in some cases (Matthies et al., 1996; Skold et al., 2005). Ncd also promotes pole coalescence in acentrosomal *Drosophila* S2 cells (Goshima et al., 2005; Ito and Goshima, 2015), suggesting that these minus-end directed kinesins may have conserved roles in pole assembly. Like KLP-15/16, Ncd is localized throughout oocyte meiosis I spindles (Hatsumi and Endow, 1992; Mullen and Wignall, 2017), and the ability of kinesin-14 family members to cross-link parallel microtubules might contribute to pole coalescence (Fink et al., 2009).

With respect to the pole instability caused by loss of ZYG-9 or TAC-1, both also promote microtubule instability during oocyte meiosis I, raising the possibility that excessive microtubule growth might disrupt pole coalescence. In *Drosophila*, the ZYG-9 and TAC-1 orthologs Minispindles and D-TACC also are enriched at oocyte meiotic spindle poles, and loss of their function often results in tripolar spindles (Cullen and Ohkura, 2001), although the dynamics of pole stability have not been reported. Moreover, *Drosophila* Ncd is required for Minispindles to localize to oocyte meiotic spindle poles, and thus this minus-end directed kinesin-14 family member has been proposed to transport Minispindles to oocyte spindle poles and thereby promote pole assembly.

Finally, ZYG-9 can promote microtubule assembly *in vitro* when incorporated into a centrosomal matrix that undergoes phase transitions (Woodruff et al., 2017), and the mouse XMAP215 and TACC orthologs chTOG and TACC3 have been reported to undergo phase transitions during mouse oocyte meiotic spindle assembly (So et al., 2019). Given the broad distribution of ZYG-9 and its binding partner TAC-1 during *C. elegans* oocyte meiotic spindle assembly, this protein complex may undergo phase transitions that influence microtubule and spindle pole stability. Future studies that assess in more detail the dynamics of ZYG-9/TAC-1 during oocyte meiosis I, and how ZYG-9/TAC-1 and KLP-15/16 interact, should further advance our understanding of how these regulators contribute to the assembly of bipolar but acentrosomal oocyte meiotic spindles.

## MATERIALS AND METHODS

### *C. elegans* strains

*C. elegans* strains used in this study are listed in Table S1. All strains were maintained at 20°C on standard nematode growth medium plates seeded with *E. coli* strain OP50.

### RNAi

All RNAi experiments were carried out by feeding *E. coli* strain HT115(DE3) induced to express double-stranded RNA corresponding to each gene as previously described (Kamath et al., 2001; Timmons and Fire, 1998). The bacteria clones were picked from an RNAi library (Kamath et al., 2003). Synchronized L1 stage larvae were grown on standard nematode growth medium plates until designated time, washed with M9 three times and then plated on the induced plates and grown at 20°C until imaging. The time that worms were fed on the induced plates varied from gene to gene in order to achieve maximum gene reduction without causing sterilization. For *tac-1*, worms were fed for 96–100 h; for *tbg-1* and *zyg-9*, 48–52 h; for *lmn-1*, 24–28 h and for *ran-1*, 16–20 h. The feeding times were chosen such that if treatment were extended for 6 more hours, 90% or more of the adult worms became sterile, with one imaging session during which worms were mounted for *in utero* movies lasting 4–6 h. We also scored embryonic lethality from worms isolated at the same time from the same plates that we used to collect worms for whole mount imaging of oocytes; in all cases we observed 99–100% embryonic lethality. To further verify strong knockdowns, we observed the first (P_0_) mitotic cell division in one-cell stage embryos from the same worms used for imaging oocyte meiotic cell division (embryos present in the uteri of the wholemount worms), and we only used data from oocyte imaging if we observed previously published strong mitotic defects: NEBD prior to egg and sperm pronuclei meeting or abnormal P_0_ spindle assembly for LMN-1 (16 of 20 embryos); lack of chromosome condensation and P_0_ chromosome segregation defects for RAN-1 (19 of 20 embryos); failure to assemble a bipolar P_0_ mitotic spindle for TBG-1 (18 of 20 embryos); transverse P_0_ mitotic spindle for ZYG-9 (17 of 20 embryos). In *klp-15(ok1958) klp-16(or1952)* oocytes, we did not characterize any mitotic defects but observed multiple egg pronuclei in 62.5% of mitotic one-cell stage embryos (10 of 16 embryos), with a single egg pronucleus in the other six embryos.

### CRISPR

#### Generation of *zyg-9* and *tac-1* transgenic strains

The appropriate sgRNA and PAM sites for *zyg-9* were selected by using the website http://crispr.mit.edu/. The repair oligo for *zyg-9* was obtained by asymmetric PCR using primers containing flanking bases at both the 5′ and 3′ ends of the PAM site to amplify the GFP-coding region from pCFJ150-GFP(dpiRNA)::CDK-1 (Zhang et al., 2018); Addgene plasmid#107938). The injection mixture of *zyg-9* repair oligo, co-CRISPR marker *dpy-10* repair oligo (Arribere et al., 2014, IDT), *dpy-10* crRNA (GCTACCATAGGCACCACGAG, IDT), trRNA (IDT) and Cas9-NLS nucleases (IDT) were injected into wild-type N2 young adults. The F1 progeny of the injected animals were selected for the roller phenotype and screened for GFP expression. The non-roller/dumpy F2 progeny of the F1 animals with correct GFP expression were identified and then further outcrossed with N2.

The transgenic strain of *tac-1* was made by the same approach as described above but with *tac-1* repair oligo and *tac-1* crRNA(CAACACAACCTTCACCAAAG, IDT).

#### Generation of double deletion strain of *klp-15* and *klp-16*

The *klp-15(ok1958) klp-16(or1952)* double mutant strain was generated by the same approach as described above, with a few modifications. The injection mix was injected into *klp-15(ok1958)* single mutants, with *klp-16* crRNA(TACTATCGGAGCACCGCCGA, IDT), and no repair template was provided. Injected hermaphrodites were kept at 15°C, and their broods were screened for *dpy-10* roller or dumpy co-conversion worms. Broods produced by hermaphrodites with the *dpy-10* co-conversion marker were screened for potential *klp-15*/*16* double mutant phenotypes (embryonic lethality), and lines identified as possibly carrying mutations to both *klp-15* and *klp-16* were balanced and Sanger sequenced after PCR amplification to identify the CRISPR/Cas9 induced mutation. The *in situ* fusion of GFP to the endogenous *aspm-1* locus in the *klp-15(ok1958) klp-16(or1952)* background was made by SunyBiotech using CRISPR/Cas-9 with the allele designation *aspm-1(syb1260)*.

### Image acquisition

*In utero* filming of oocytes was accomplished by mounting young adult worms with single row of embryos on a 5% agarose pad, with 1.5 µl each of M9 buffer and 0.1 µm polystyrene microspheres (Polysciences Inc.) on a microscope slide covered with a coverslip. *Ex utero* filming of oocytes for data in Fig. S16A and Movies 10 and 11 was done by cutting open young adult worms in 3 µl of egg buffer (118 mM NaCl, 48 mM KCl, 2 mM CaCl_2_, 2 mM MgCl_2_, and 25 mM HEPES, PH 7.3) on a coverslip before mounting onto a 2% agarose pad on a microscope slide; all other data is from *in utero* imaging. Nomarski images were acquired on AxioSkop compound microscope (Zeiss) equipped with CCD camera using ImageJ software (National Institutes of Health). Fluorescence imaging was performed at room temperature (22–23˚C) using a Leica DMi8 microscope outfitted with a spinning disk confocal unit – CSU-W1 (Yokogawa) with Borealis (Andor), dual iXon Ultra 897 (Andor) cameras, and a 100x HCX PL APO 1.4-0.70NA oil objective lens (Leica). Metamorph (Molecular Devices) imaging software was used for controlling image acquisition. For *in utero* movies of oocytes from GFP::LMN-1, mCherry::H2B and GFP::TAC-1, mCherry::H2B strains, the 488 nm and 561 nm channels were imaged simultaneously every 10 s with 1 µm Z-spacing; every 5 s for oocytes from all other transgenic strains expressing fluorescent markers. 14 focal planes/z-stack were collected for all *klp-15/16* mutant oocytes and the control oocytes expressing GFP::TBB-2, mCherry::H2B in Fig. 6; 21 focal planes/z-stack were collected for all other oocytes. Time lapse images in figures depict maximum projections for all fluorescent proteins except for GFP::LMN-1, which were single focal planes of maximum oocyte diameters. Time lapse supplemental movies show maximum projections for all z-stack focal planes unless otherwise indicated.

### Image processing and analysis

General imaging process, including merging red/green channels, cropping, stabilizing and z-projected images, was performed with ImageJ software (National Institutes of Health). Three-dimensional projection and rotation movies were made by using Imaris software (Bitplane).

Normalized microtubule pixel intensity quantification was carried out by ImageJ software. The spindle area was determined by generating auto-threshold (set on Otsu) in a cropped area in the microtubule channel around the spindle in the projected z stack (Max intensity) treated with Gaussian blur (sigma set at 2) at time points throughout meiosis I. The regions of interest (ROIs) were selected accordingly and saved in ROI manager. Measurements of microtubule intensity were taken by applying the saved ROIs to the projected z-stack (sum slices) in the microtubule channel at the corresponding time points. Both the area of the ROIs and the mean gray value (MeanGV) were automatically calculated. In addition, an area excluding the spindle was selected and the MeanGV was calculated for the cytoplasm. Measurements of chromosome intensity were taken by selecting the oocyte chromosome area in the histone channel in the projected z-stack (Max intensity) at the corresponding time points and the maximum gray value (MaxGV) were calculated. Additionally, an area excluding the oocyte and the sperm chromosomes was selected and the MaxGV was calculated for the cytoplasm. All of the measurements were placed into the following formula: [MeanGV (spindle)-MeanGV (cytoplasm)]/[MaxGV (chromosomes)-MaxGV (cytoplasm)]×area(spindle)=normalized microtubule pixel intensity.

Measurements of area occupied by chromosomes in projected z-stack were carried out by ImageJ software as described in Connolly et al., 2014.

### Statistics

*P*-values comparing distributions for all scatter plots were calculated using the Mann–Whitney *U*-test. *P*-values comparing variance for all scatter plots were calculated using the *F*-test.

## Supplementary Material

Supplementary information
