# Supplementary material for: Microtubule assembly and pole coalescence: early steps in Caenorhabditis elegans oocyte meiosis I spindle assembly
Source: Biol Open. 2020 Jun 25;9(6):bio052308. doi: 10.1242/bio.052308 (PMC7328010; doi:10.1242/bio.052308)
Supplement: Supplementary information [file biolopen-9-052308-s1.pdf]

Supp. Figure 1

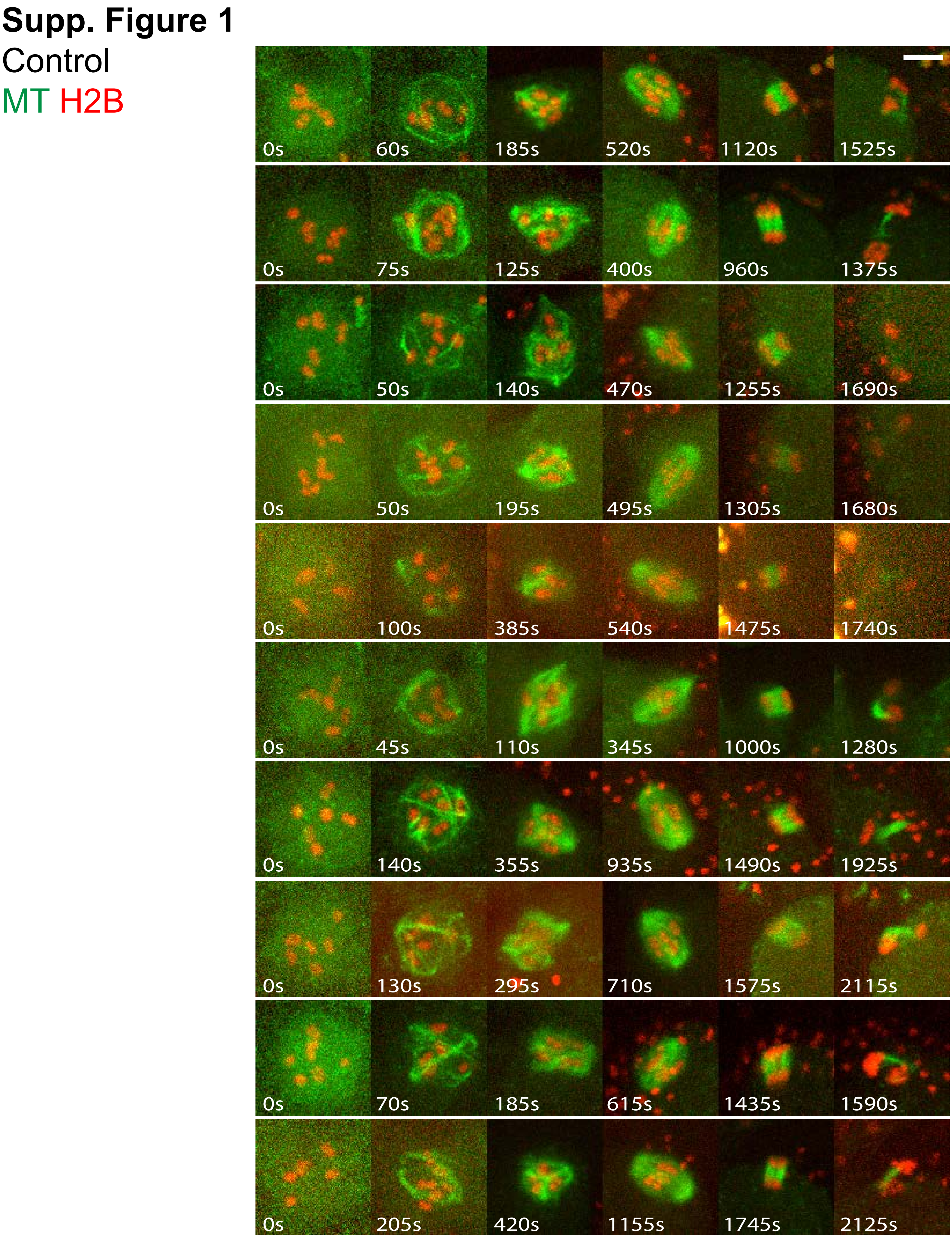

Figure S1.

Time-lapse maximum projection images during meiosis I for 10 live control oocytes expressing GFP::TBB-2 and mCherry::H2B.

Supp. Figure 2

Control
ASPM-1
H2B

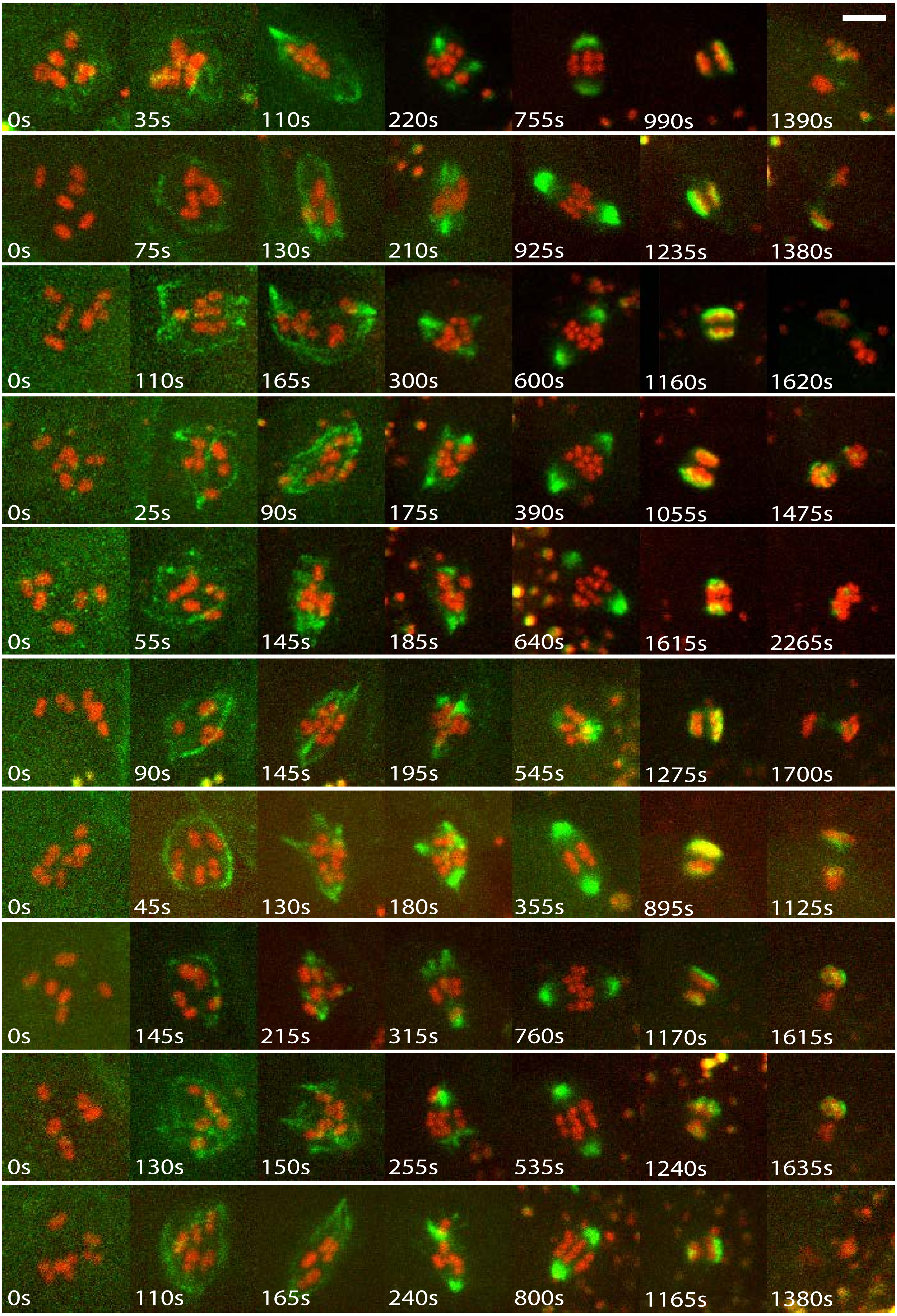

Figure S2.

Time-lapse maximum projection images during meiosis I for 10 live control oocytes expressing GFP::ASPM-1 and mCherry::H2B.

Supp. Figure 3

lmn-1(RNAi)

MT H2B

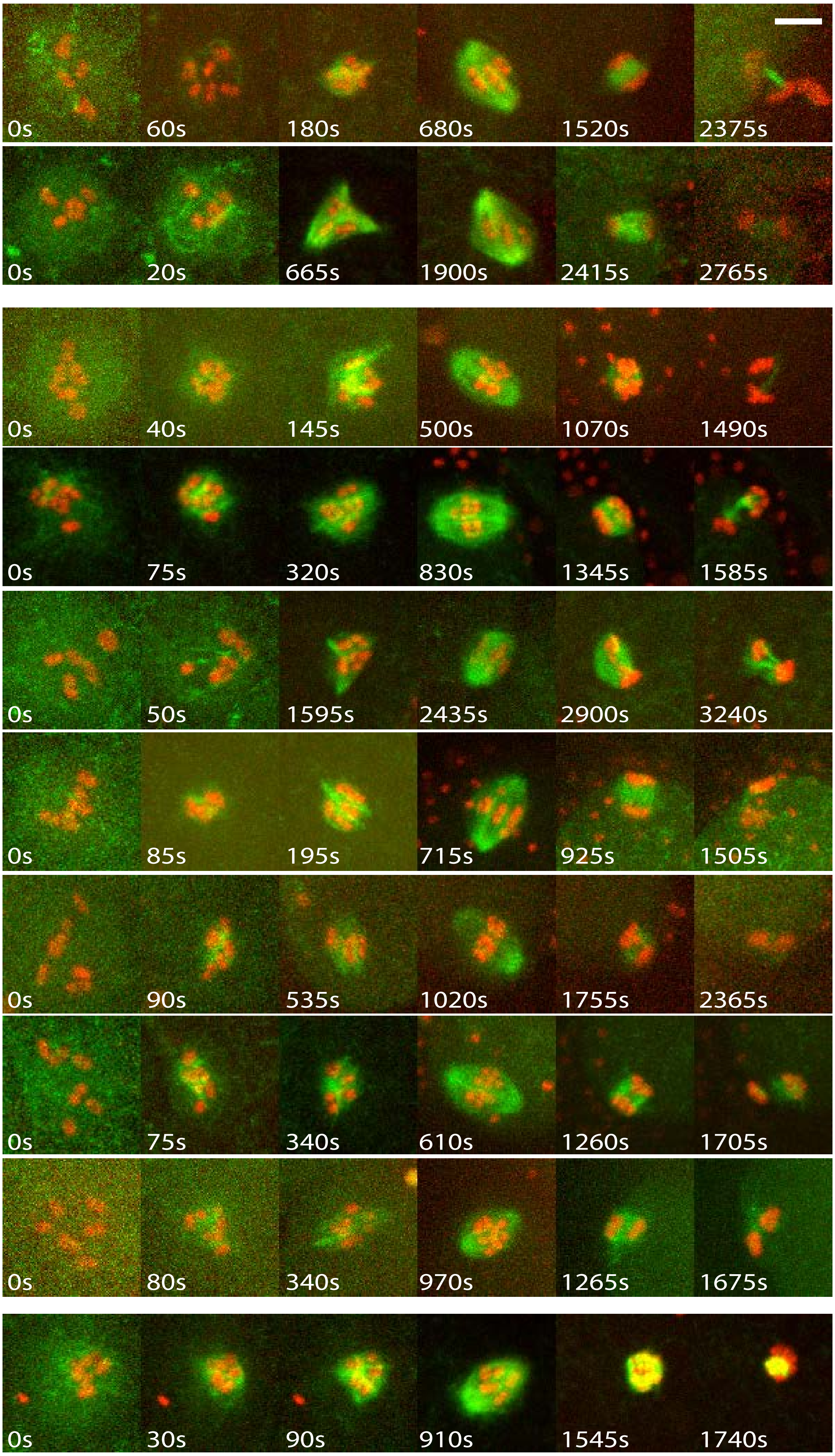

Biology Open • Supplementary information

Figure S3.

Time-lapse maximum projection images during meiosis I for 10 live *lmn-1(RNAi)* oocytes expressing GFP::TBB-2 and mCherry::H2B. Rows 1 & 2: oocytes with cage structure; Rows 3-9 oocytes without cage structure; Row 10: oocyte without cage and no chromosome segregation. In all figures, cage structures and chromosome segregation we are assessed using Imaris software to rotate 3-D images.

Biology Open • Supplementary information

Supp. Figure 4

lmn-1(RNAi)

ASPM-1 H2B

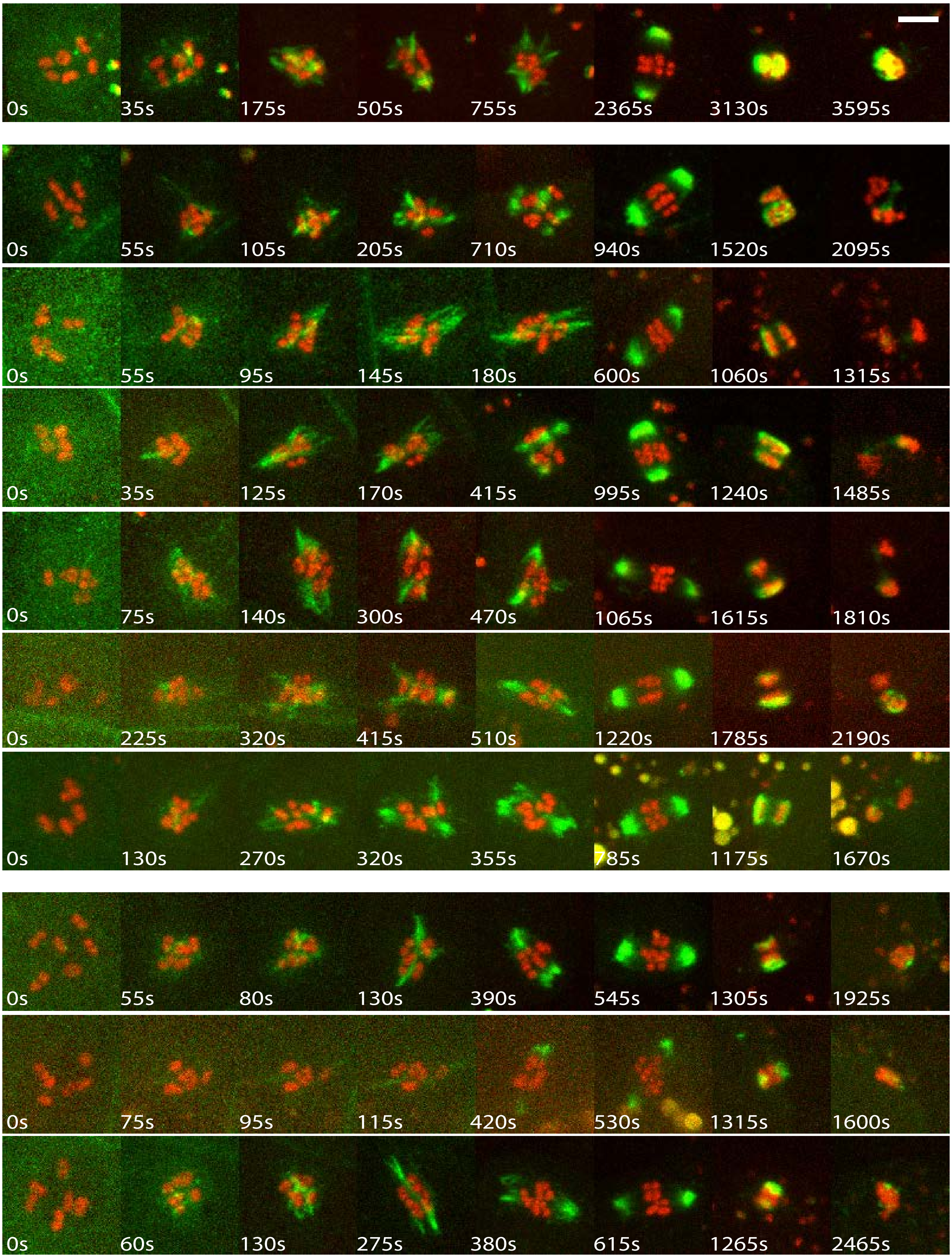

Biology Open • Supplementary information

Figure S4.

Time-lapse maximum projection images during meiosis I for 10 live *lmn-1(RNAi)* oocytes expressing GFP::ASPM-1 and mCherry::H2B. Row 1: oocyte with cage structure and no chromosome segregation; Rows 2-7 oocytes without cage structure; Row 8-10: oocytes without cage and no chromosome segregation.

Supp. Figure 5

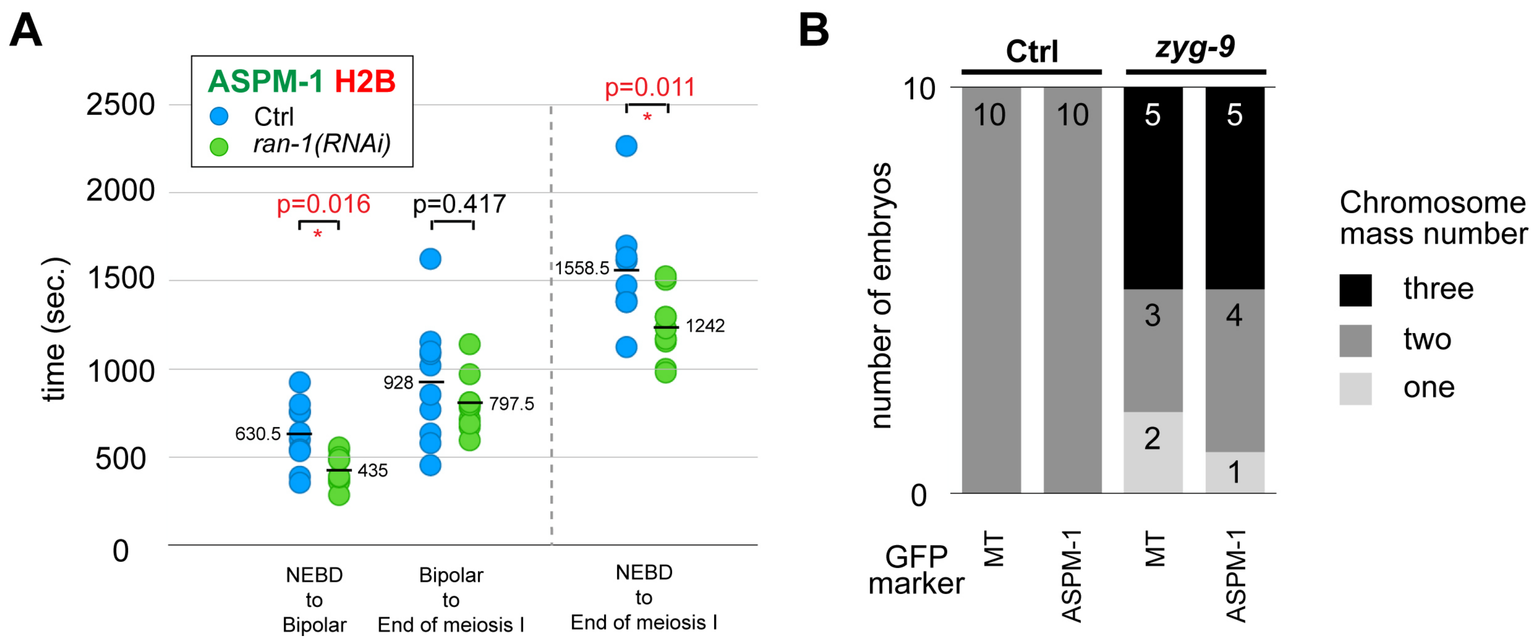

Figure S5.

(A) Scatter plot showing comparisons of the time from NEBD to spindle bipolarity establishment (Bipolar), from spindle bipolarity establishment to the end of meiosis I, and entire time to progress through meiosis I between control and *ran-1(RNAi)* oocytes expressing GFP::ASPM-1 and mCherry::H2B.

(B) The number of segregating chromosome masses detected in control and *zyg-9(RNAi)* oocytes at the end of meiosis I for strains expressing either GFP::TBB-2 and mCherry::H2B, or GFP::ASPM-1 and mCherry::H2B. Numbers within the bars indicate the number of embryos in each category.

Biology Open • Supplementary information

Supp. Figure 6

ran-1(RNAi)

MT H2B

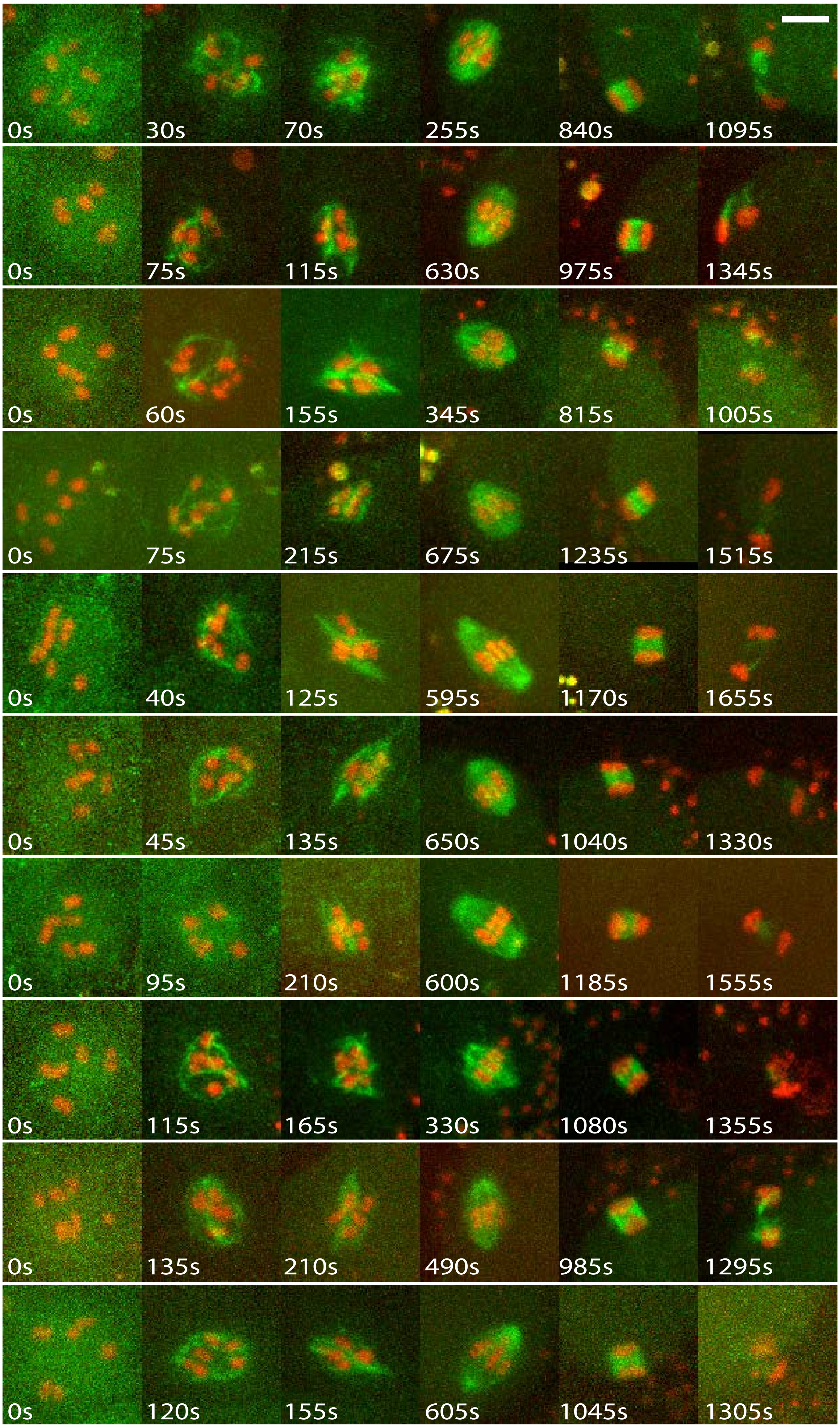

Figure S6.

Time-lapse maximum projection images during meiosis I for 10 live *ran-1(RNAi)* oocytes expressing GFP::TBB-2 and mCherry::H2B.

Biology Open • Supplementary information

Supp. Figure 7

ran-1(RNAi)

ASPM-1 H2B

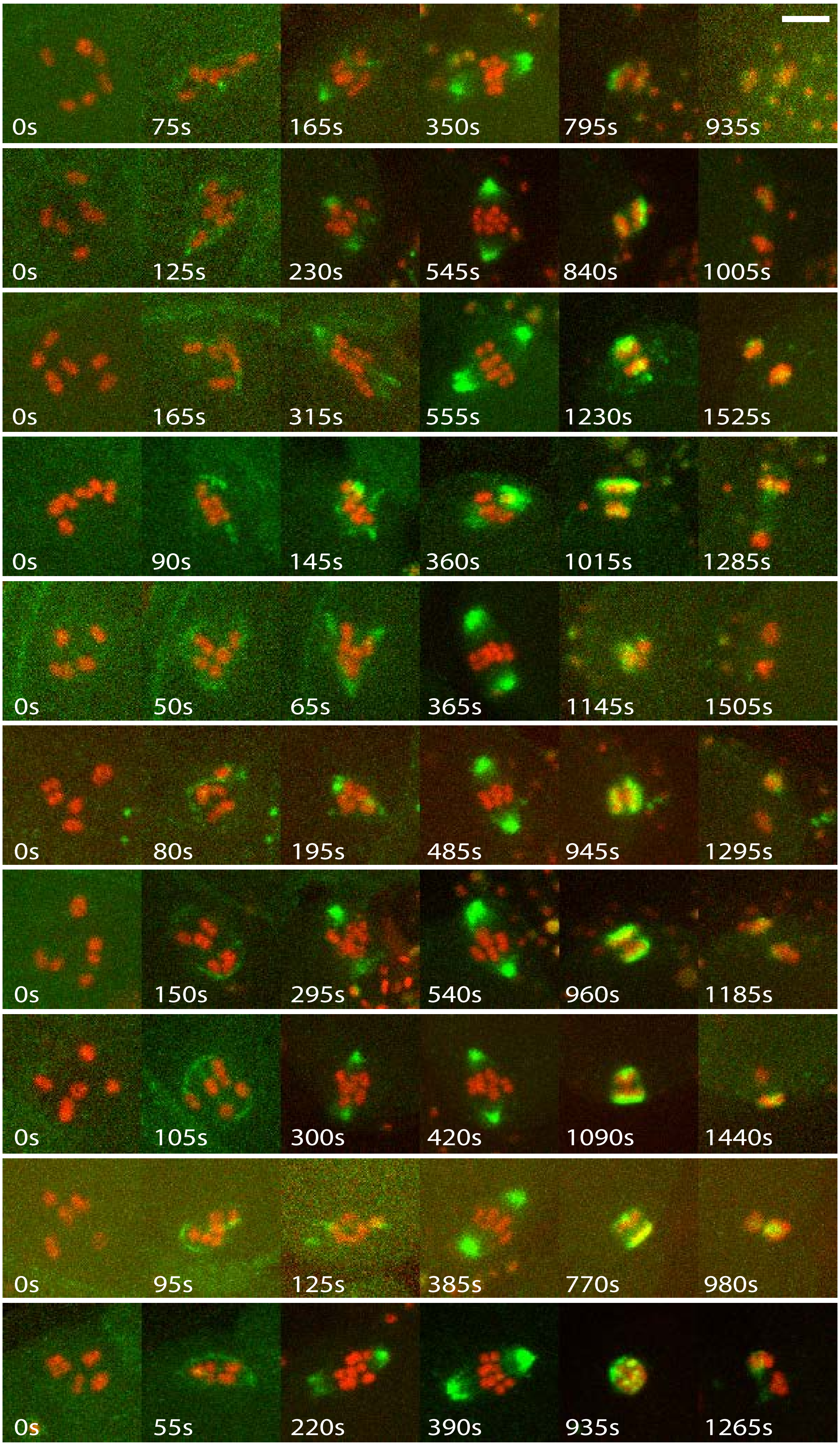

Figure S7.

Time-lapse maximum projection images during meiosis I for 10 live *ran-1(RNAi)* oocytes expressing GFP::ASPM-1 and mCherry::H2B.

Biology Open • Supplementary information

Supp. Figure 8

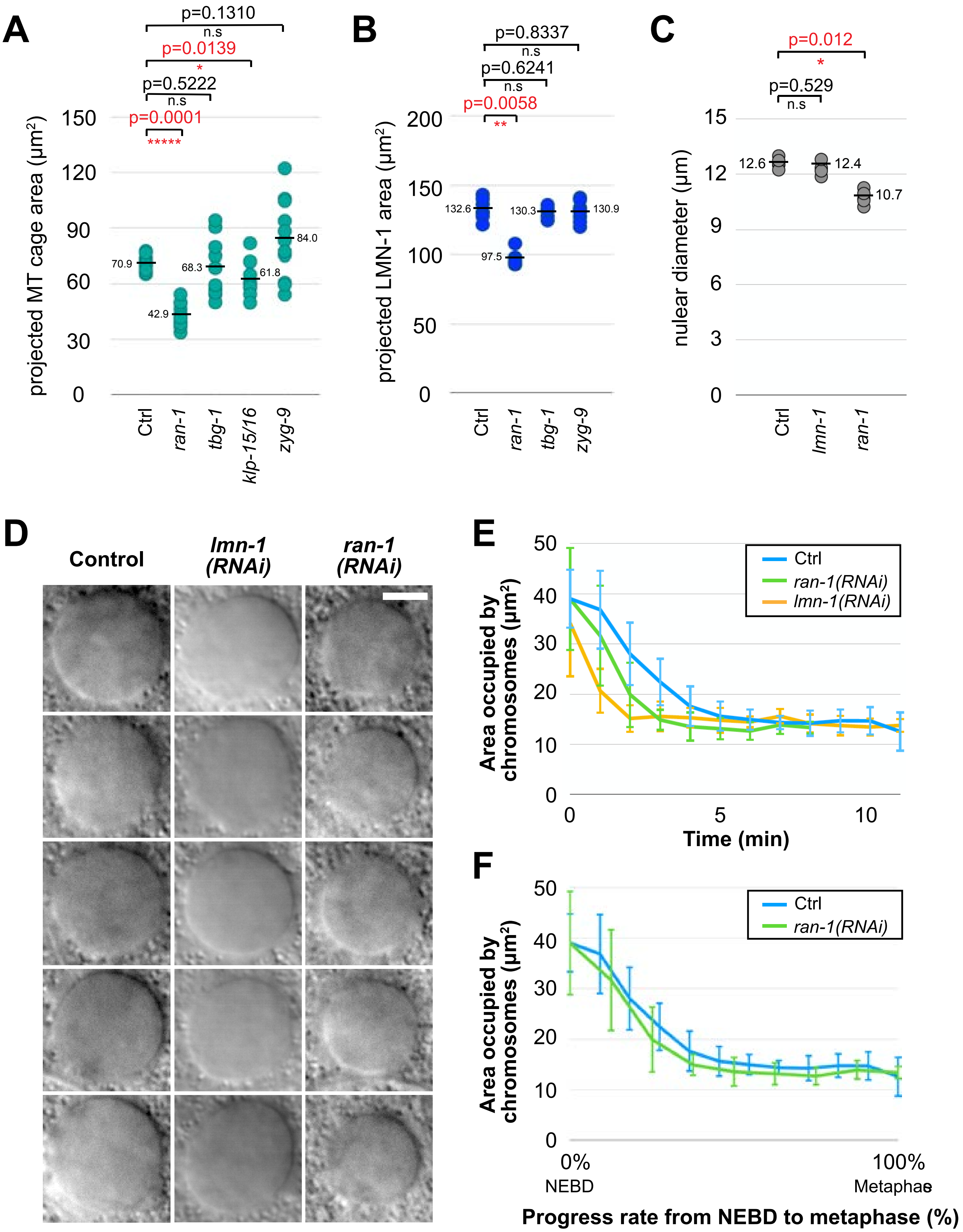

Biology Open • Supplementary information

Figure S8.

(A) Scatter plot showing projected areas of the microtubule cage-like structure meiosis I in control and mutant oocytes expressing GFP::TBB-2 and mCherry::H2B.

(B) Scatter plot showing projected nuclear lamina area measured at the cage stage of meiosis I for control and mutant oocytes expressing GFP::LMN-1 and mCherry:: TBB-2.

(C) Scatter plot showing nuclear diameter of control, *lmn-1(RNAi)* and *ran-1(RNAi)* oocytes measured using Nomarski images (8D).

(D) Nomarski images of control, *lmn-1(RNAi)* and *ran-1(RNAi)* oocyte nuclei 2 seconds before nuclear envelope break down.

(E) Area occupied by chromosomes measured from maximum projection images over time with 1-minute time intervals from NEBD to metaphase for control, *lmn- 1(RNAi)* and *ran-1(RNAi)* oocytes.

(F) Area occupied by chromosomes measured from maximum projection images plotted as percent of time from NEBD to metaphase for control and *ran-1(RNAi)* oocytes.

Biology Open • Supplementary information

Supp. Figure 9

tbg-1(RNAi)

MT H2B

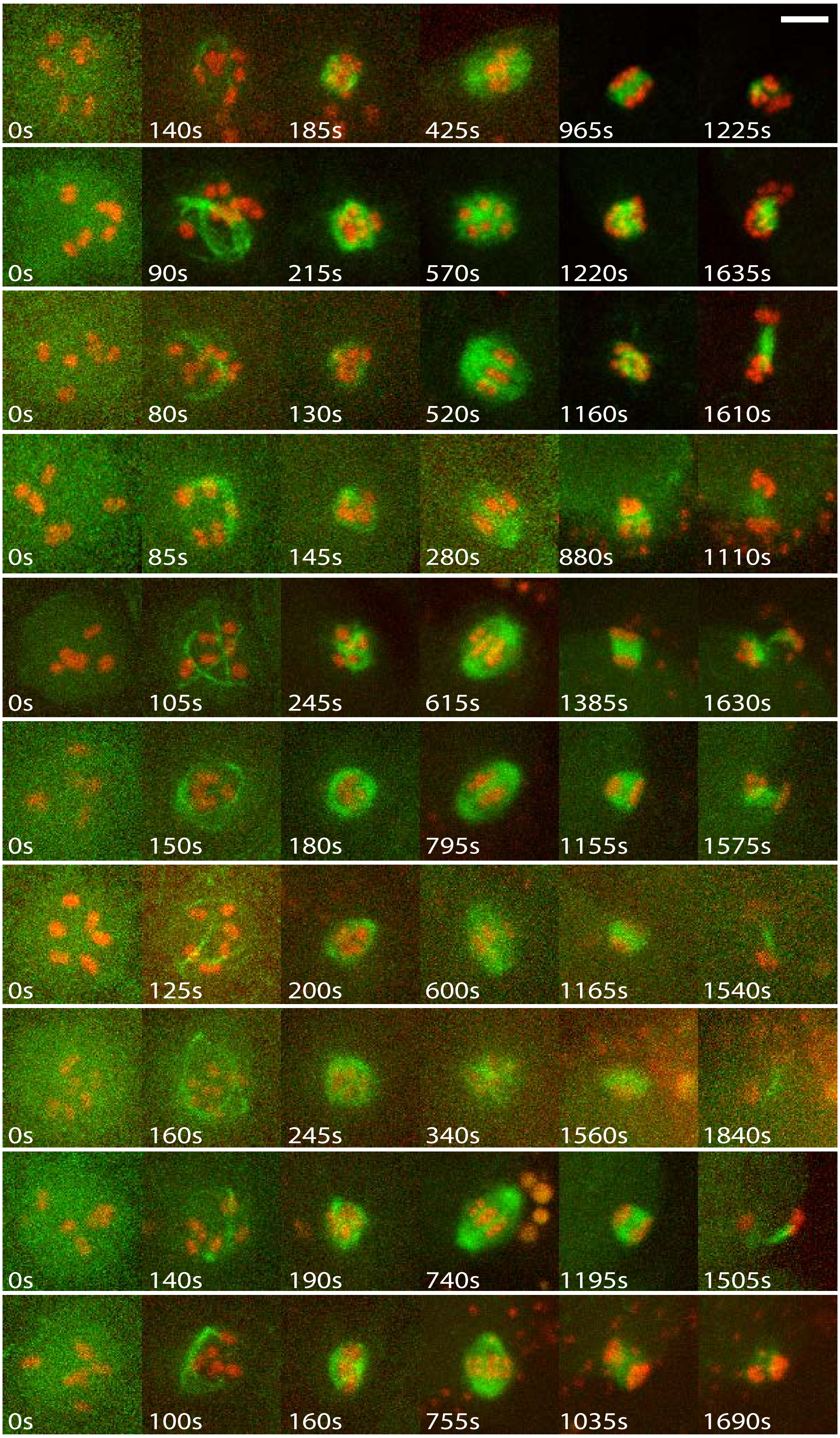

Figure S9.

Time-lapse **maximum projection i**mages during meiosis I for 10 live *tbg-1(RNAi)* oocytes expressing GFP::TBB-2 and mCherry::H2B.

Biology Open • Supplementary information

Supp. Figure 10

tbg-1(RNAi)
ASPM-1
H2B

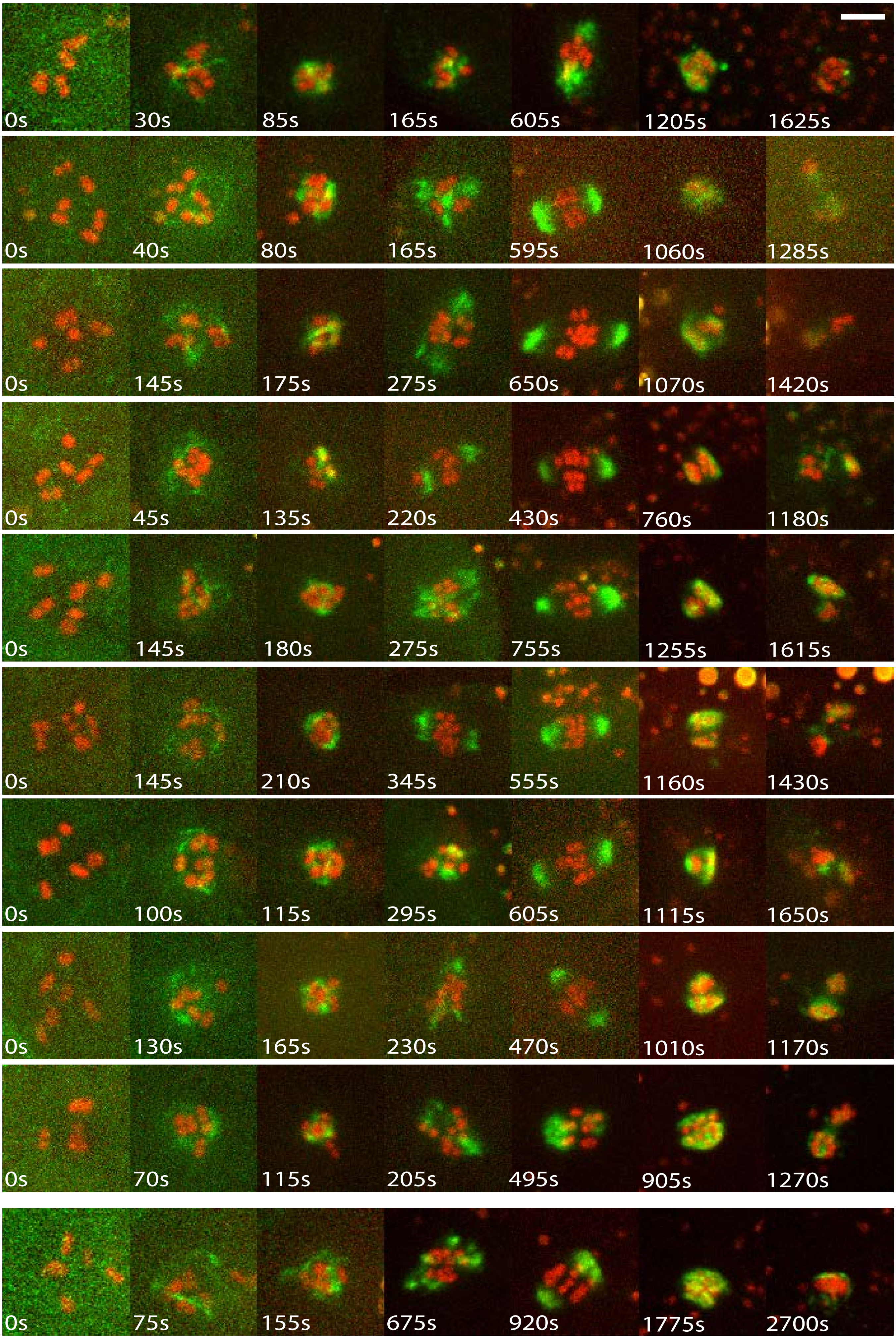

Figure S10.

Time-lapse maximum projection images during meiosis I for 10 live *tbg-1(RNAi)* oocytes expressing GFP::ASPM-1 and mCherry::H2B. Rows 1-9: oocytes with chromosome segregation; Row 10: oocyte without chromosome segregation.

Biology Open • Supplementary information

Supp. Figure 11

klp-15(ok1958) klp-16(or1952)

MT H2B

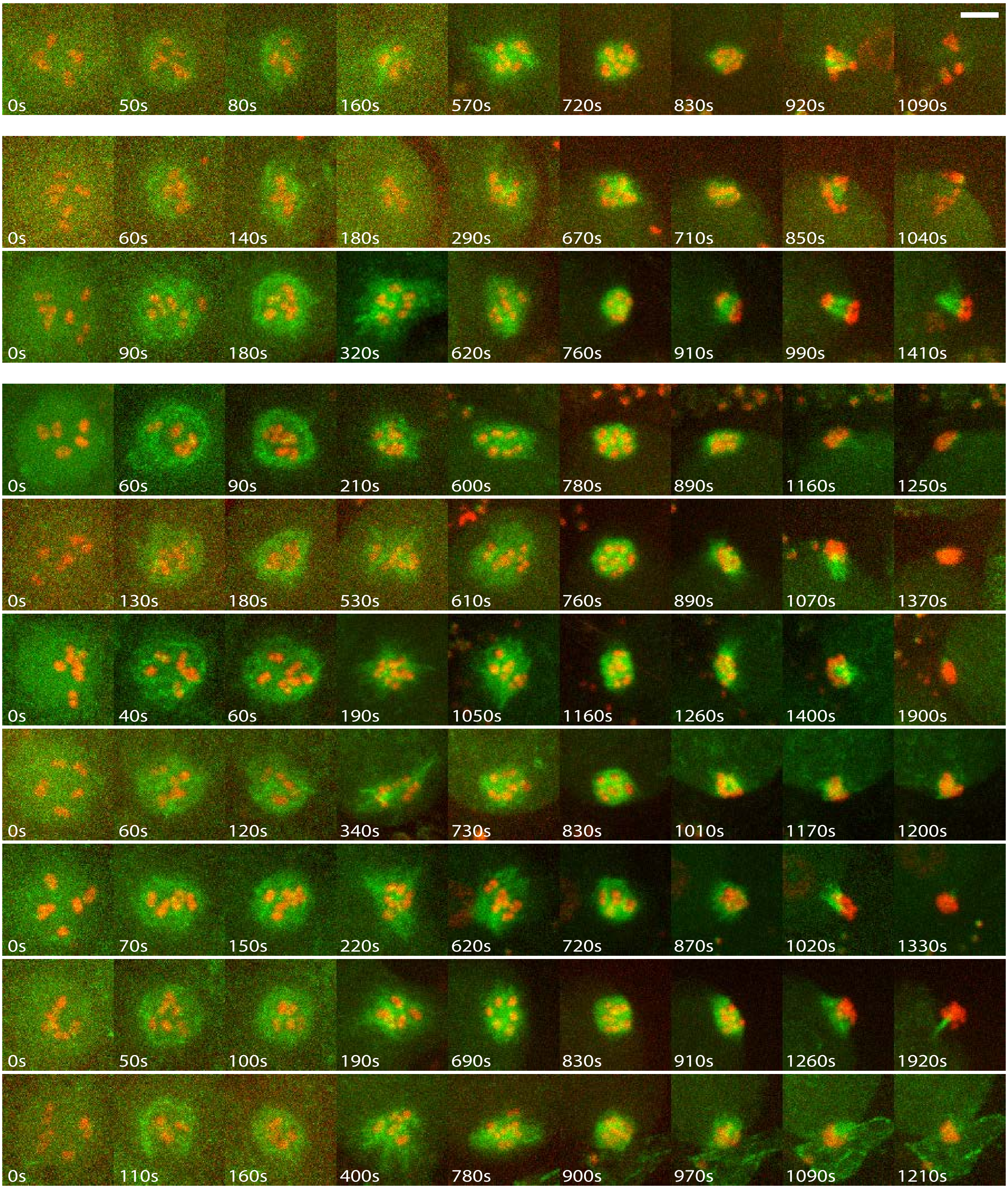

Figure S11.

Time-lapse maximum projection images during meiosis I for 10 live *klp-15/16(-/-)* double mutant oocytes expressing GFP::TBB-2 and mCherry::H2B. Row 1: oocyte with chromosomes segregated into three masses; Rows 2 & 3: oocytes with chromosomes segregated into two masses; Rows 4-10: oocytes with no chromosome segregation.

Biology Open • Supplementary information

Supp. Figure 12

klp-15(ok1958) klp-16(or1952)

ASPM-1 H2B

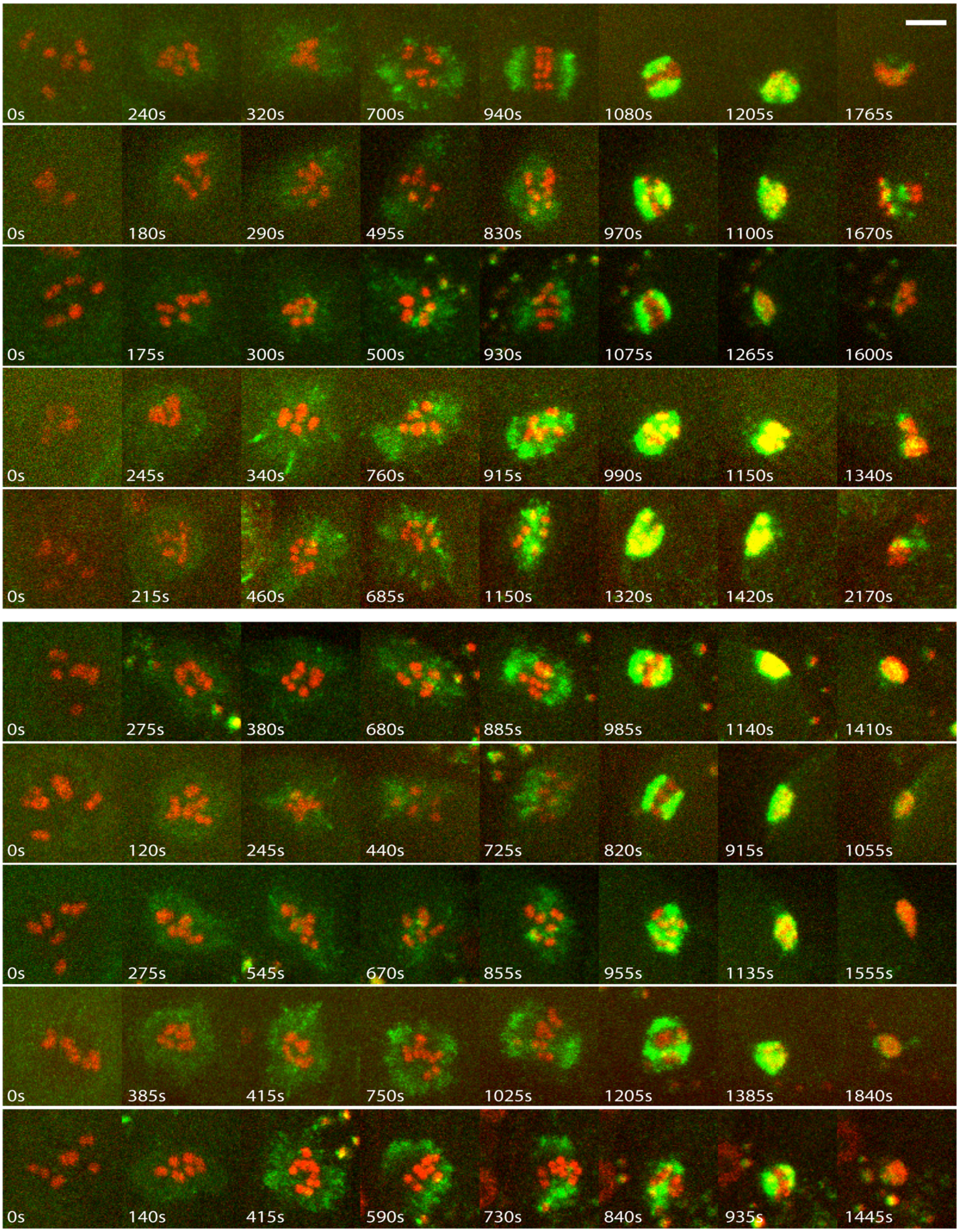

Figure S12.

Time-lapse maximum projection images during meiosis I for 10 live *klp-15/16(-/-)* double mutant oocytes expressing GFP::ASPM-1 and mCherry::H2B. Rows 1-5: oocytes with chromosomes segregated into two masses; Rows 6-10: oocytes with no chromosome segregation.

Biology Open • Supplementary information

Supp. Figure 13
zyg-9(RNAi)
MT H2B

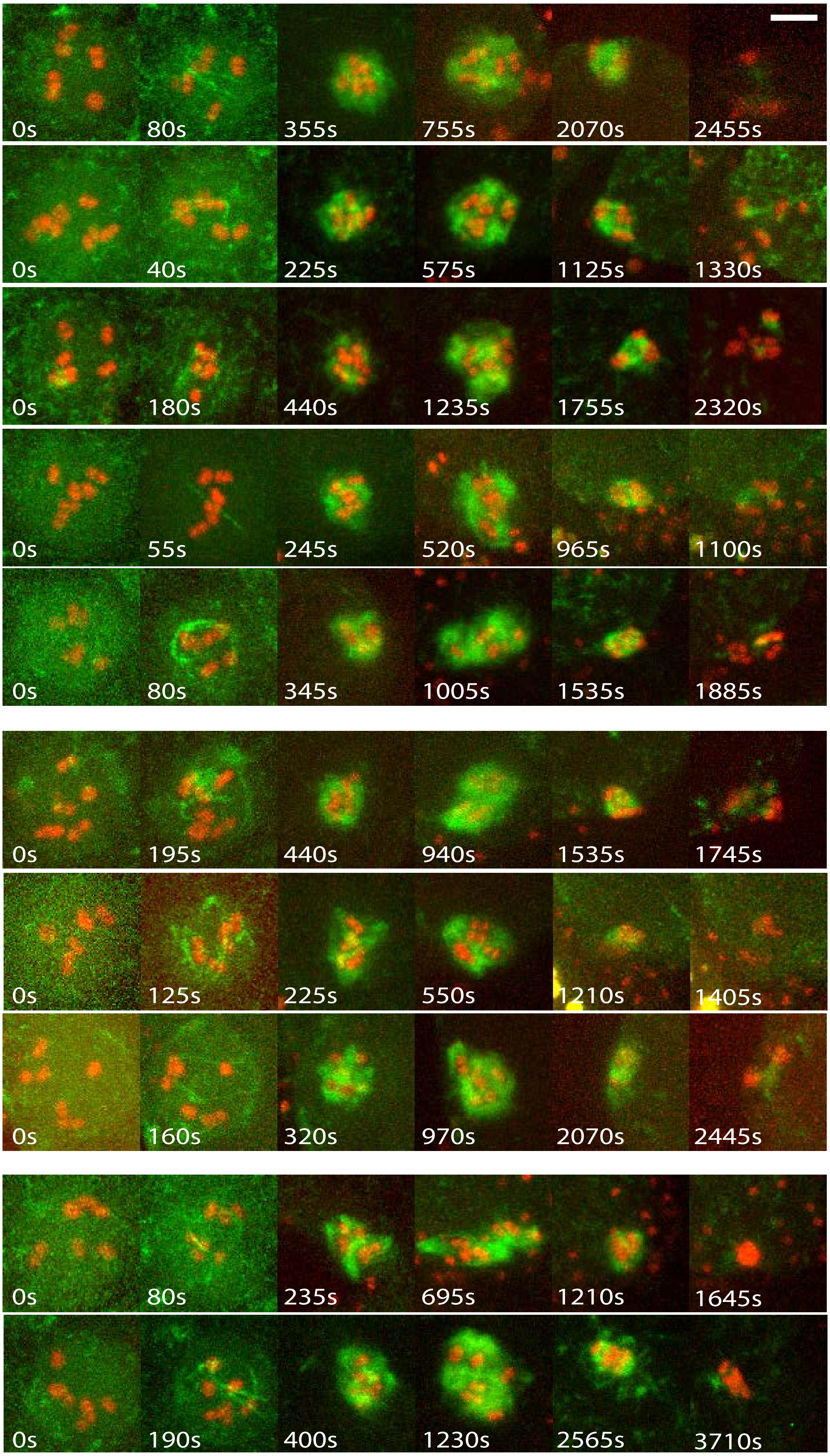

Figure S13.

Time-lapse maximum projection images during meiosis I for 10 live *zyg-9(RNAi)* oocytes expressing GFP::TBB-2 and mCherry::H2B. Rows 1-5: oocytes with chromosomes segregated into three masses; Rows 6-8: oocytes with chromosomes segregated into two masses; Rows 9 & 10: oocytes with no chromosome segregation.

Biology Open • Supplementary information

Supp. Figure 14

zyg-9(RNAi)

ASPM-1 H2B

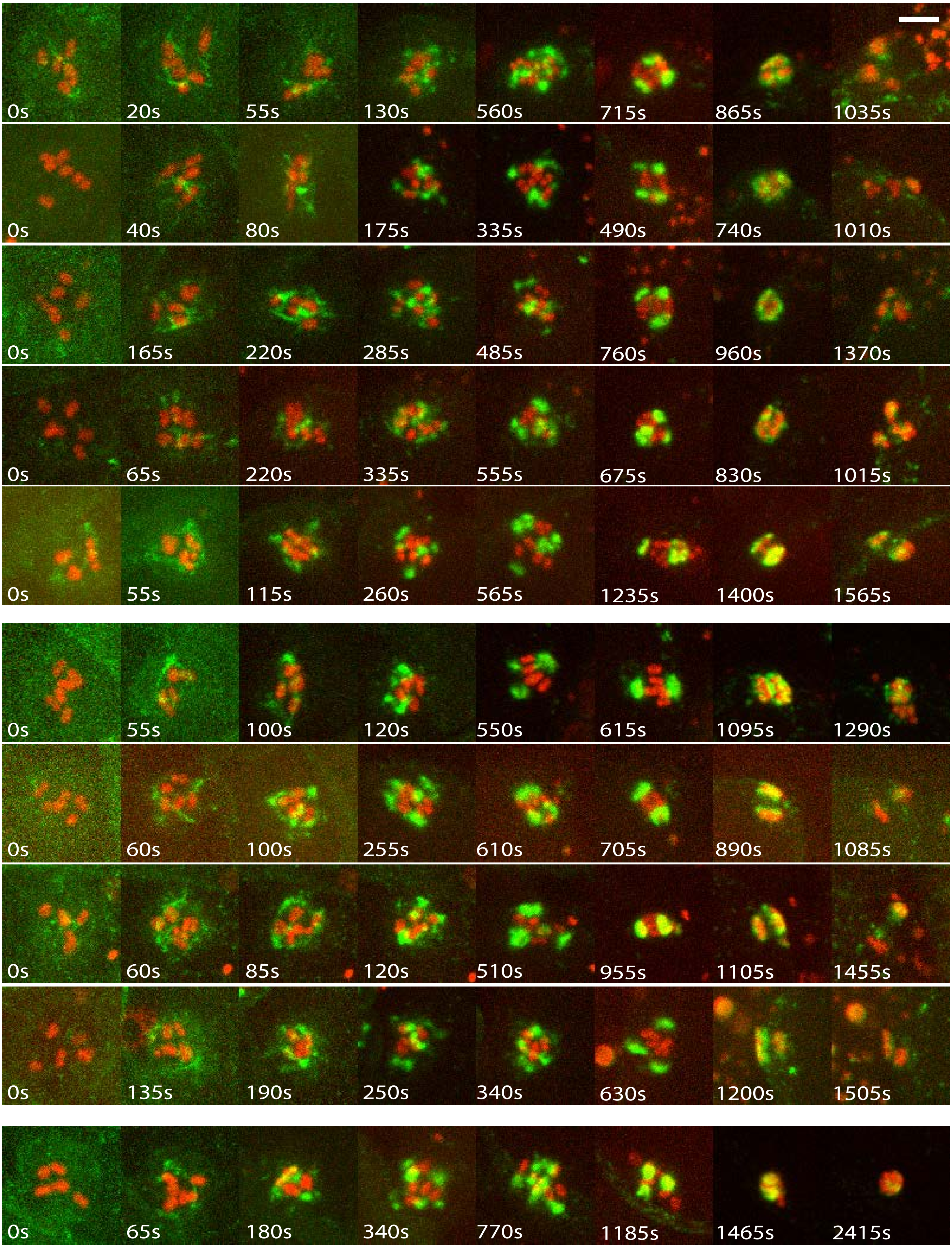

Figure S14.

Time-lapse maximum projection images during meiosis I for 10 live *zyg-9(RNAi)* oocytes expressing GFP::ASPM-1 and mCherry::H2B. Rows 1-5: oocytes with chromosomes segregated into 3 masses; Rows 6-9: oocytes with chromosomes segregated into two masses; Row 10: no chromosome segregation.

Biology Open • Supplementary information

Supp. Figure 15

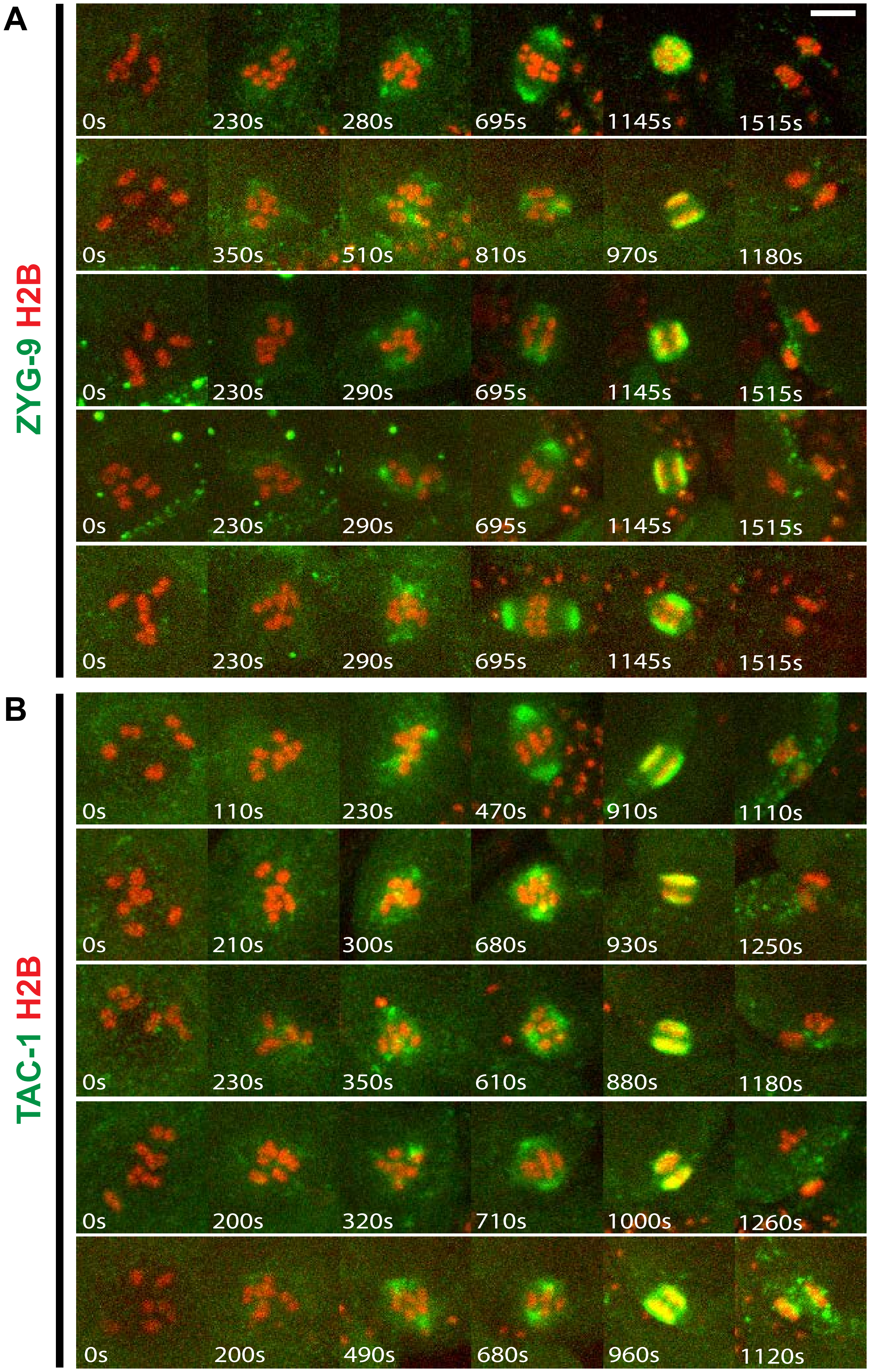

Figure S15.

Time-lapse maximum projection images during meiosis I for live control oocytes expressing either GFP::ZYG-9 and mCherry::H2B (A), or GFP::TAC-1 and mCherry::H2B (B).

Supp. Figure 16

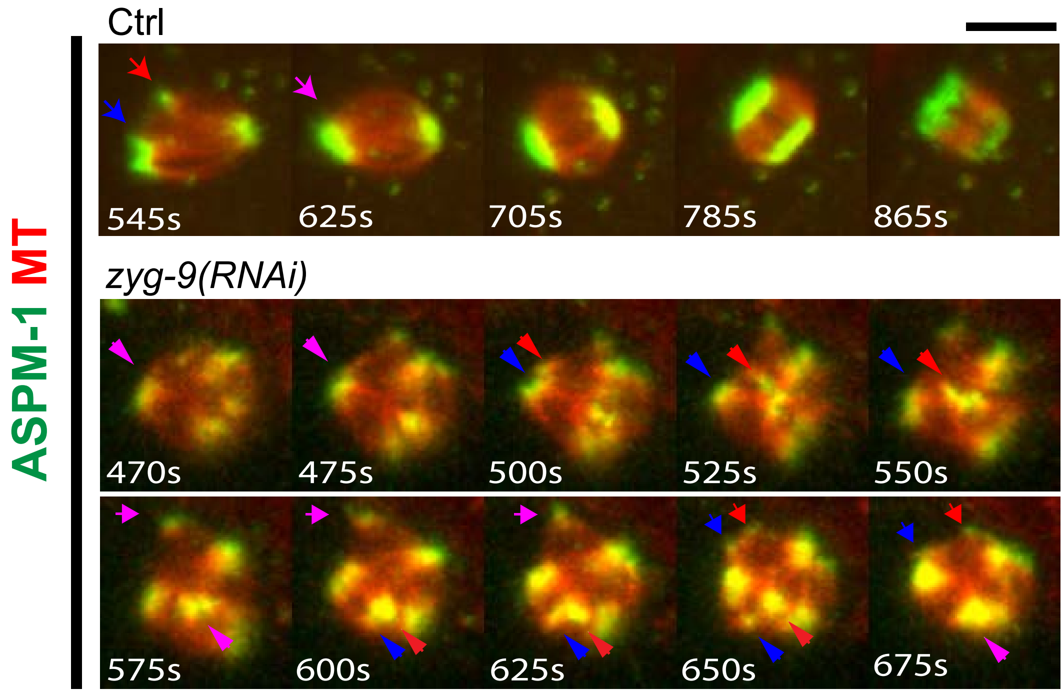

Figure S16.

Time-lapse maximum projection images during meiosis I for live control and *zyg-9(RNAi)* oocytes expressing GFP::ASPM-1 and mCherry:: TBB-2. Times after NEBD are indicated. Pink arrowheads indicate spindle poles that split into two foci that are then marked by red and blue arrowheads.

Biology Open • Supplementary information

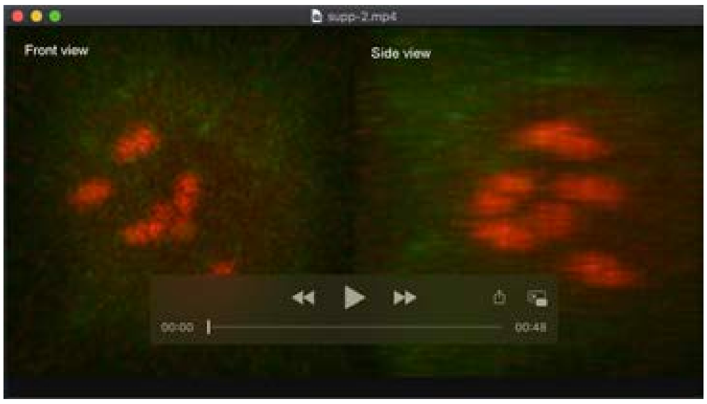

Movie 1.

Time-lapse movie during meiosis I of a live control oocyte expressing GFP::TBB-2 and mCherry::H2B.

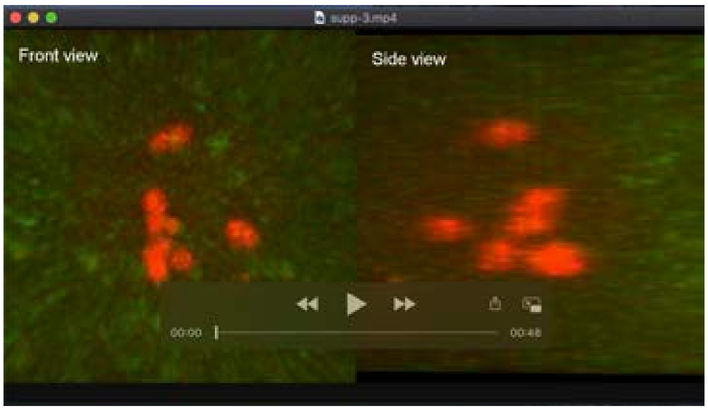

Movie 2.

Time-lapse movie during meiosis I of a live control oocyte expressing GFP::ASPM-1 and mCherry::H2B.

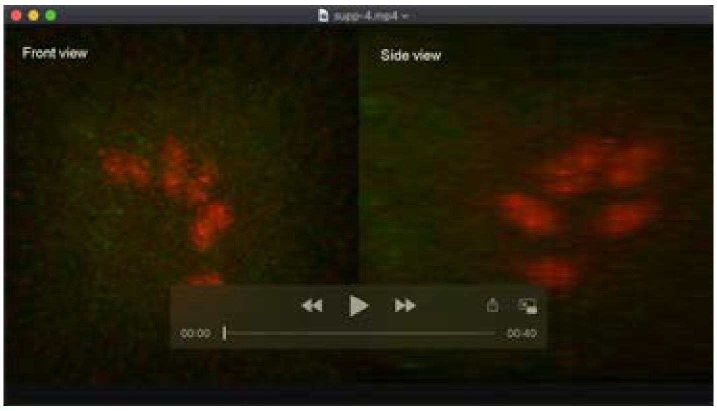

Movie 3.

Time-lapse movie during meiosis I of a LMN-1 knock-downed oocyte expressing GFP::TBB-2 and mCherry::H2B.

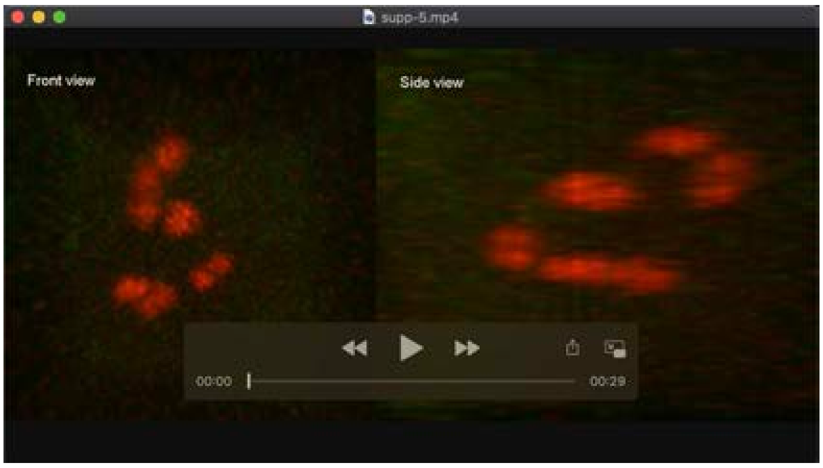

Movie 4.

Time-lapse movie during meiosis I of a LMN-1 knock-downed oocyte expressing GFP::ASPM-1 and mCherry::H2B.

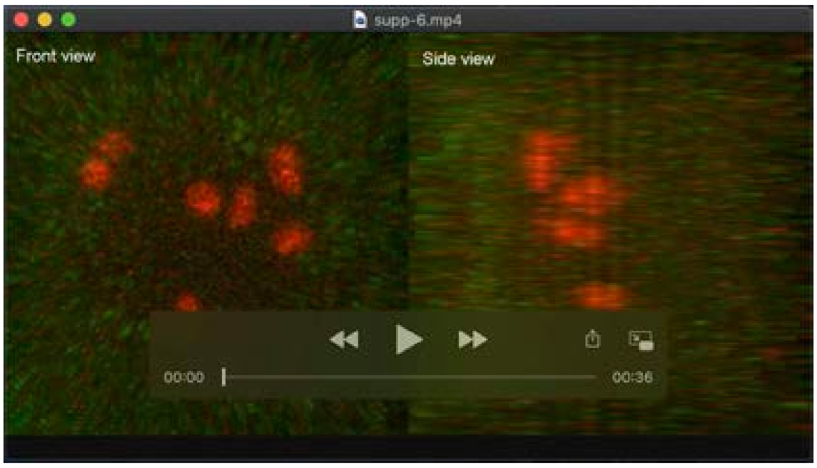

Movie 5.

Time-lapse movies during meiosis I for live *klp-15/16(-/-)* double mutant oocytes expressing GFP::ASPM-1 and mCherry::H2B.

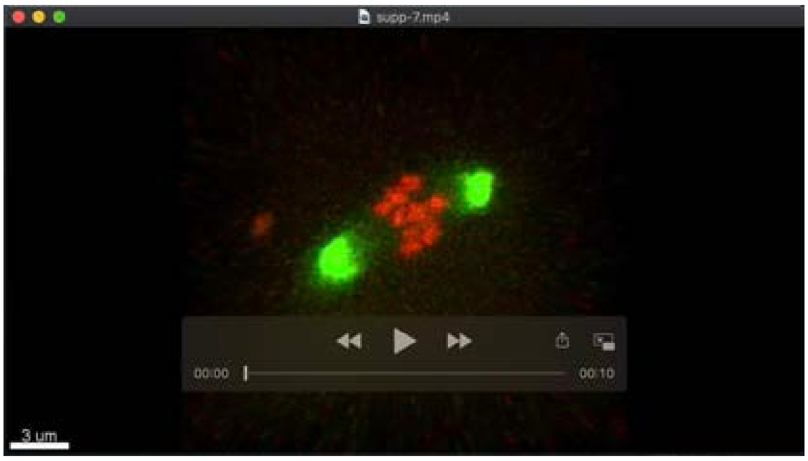

Movies 6.

Three-dimensional rotation movie at metaphase for a live control oocyte expressing GFP::ASPM-1 and mCherry::H2B.

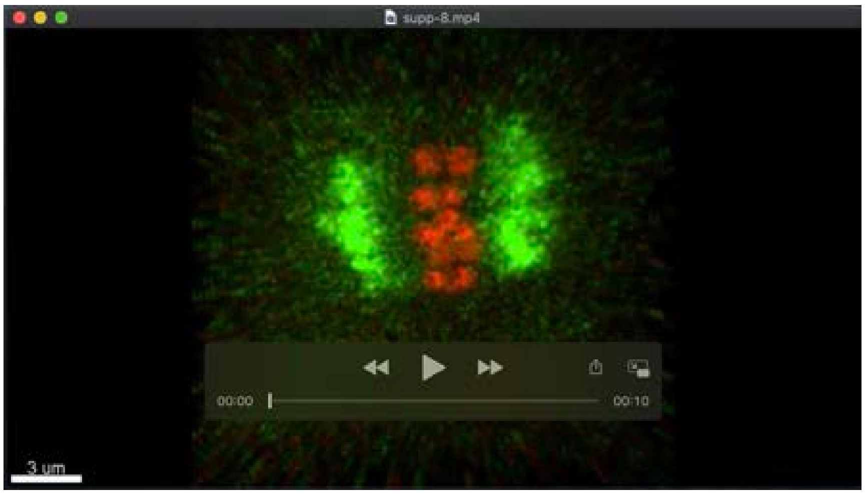

Movie 7

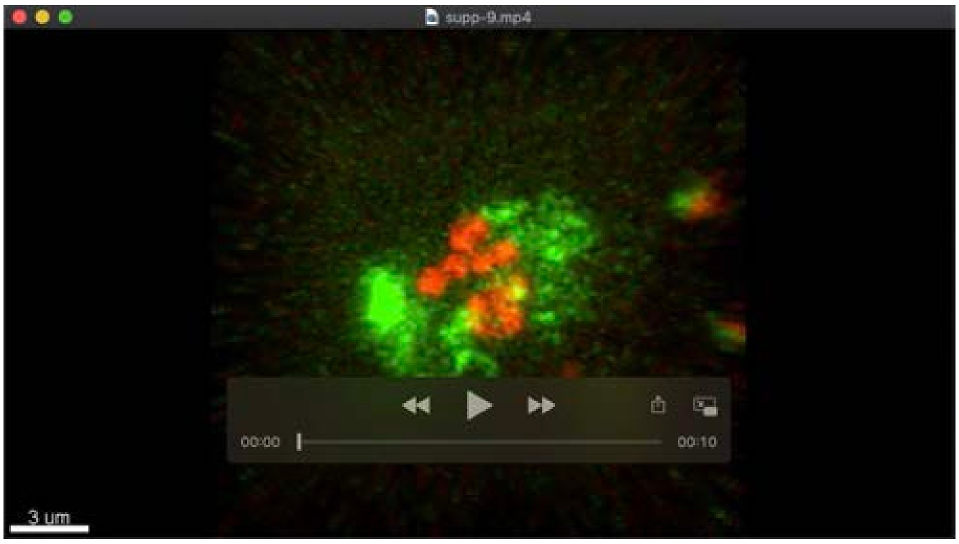

Movie 8

Movies 7 and 8.

Three-dimensional rotation movies at metaphase for live *klp-15/16(-/-)* oocytes expressing GFP::ASPM-1 and mCherry::H2B.

Biology Open • Supplementary information

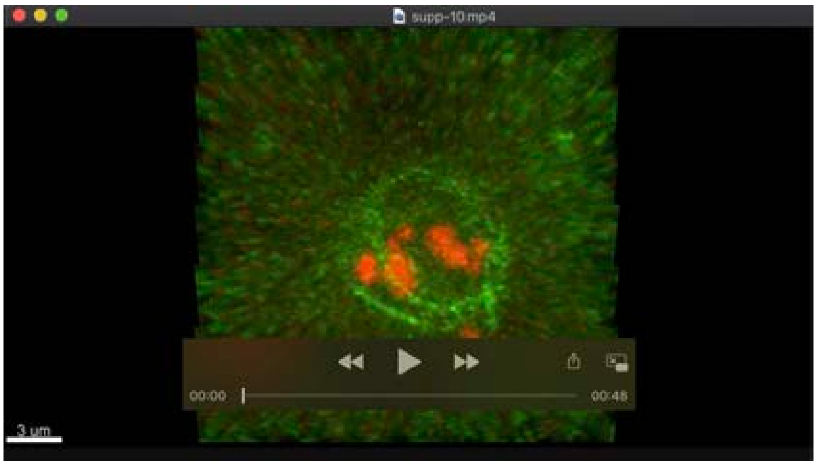

Movie 9

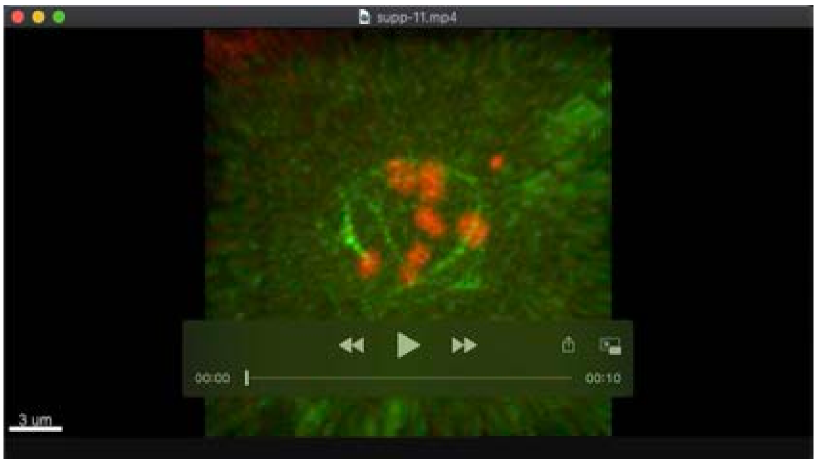

Movie 10

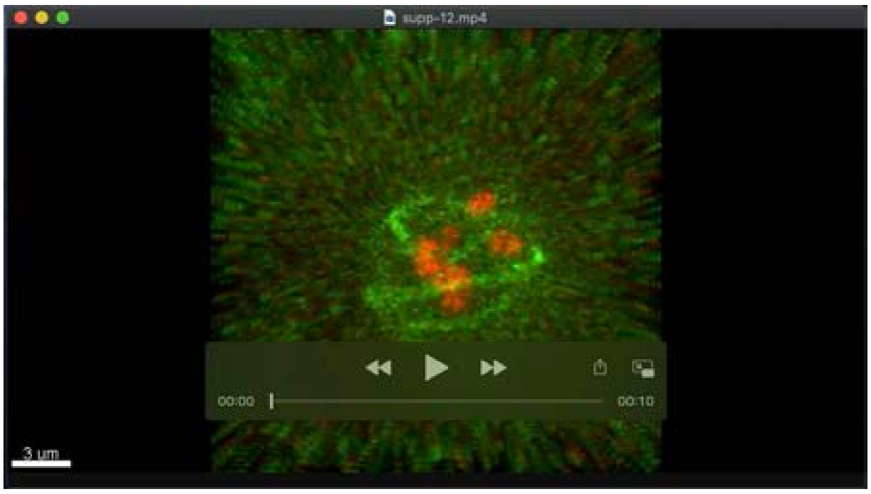

Movie 11

Movies 9, 10, and 11.

Three-dimensional rotation movies during meiosis I for live control oocytes expressing GFP::TBB-2 and mCherry::H2B.

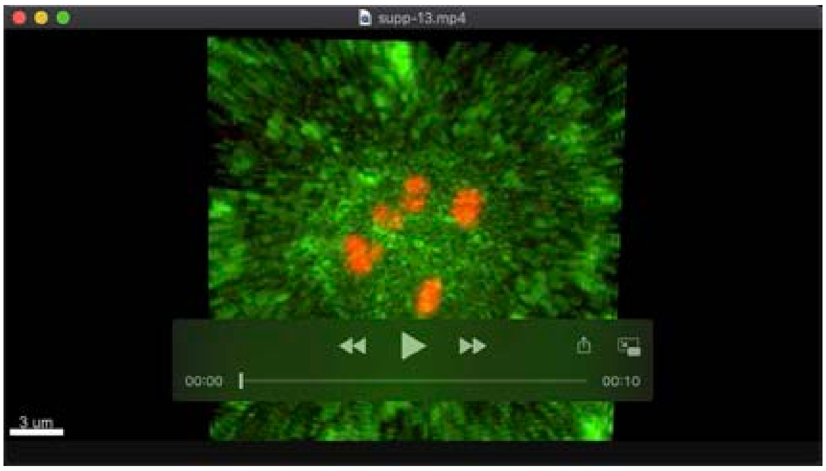

Movie 12

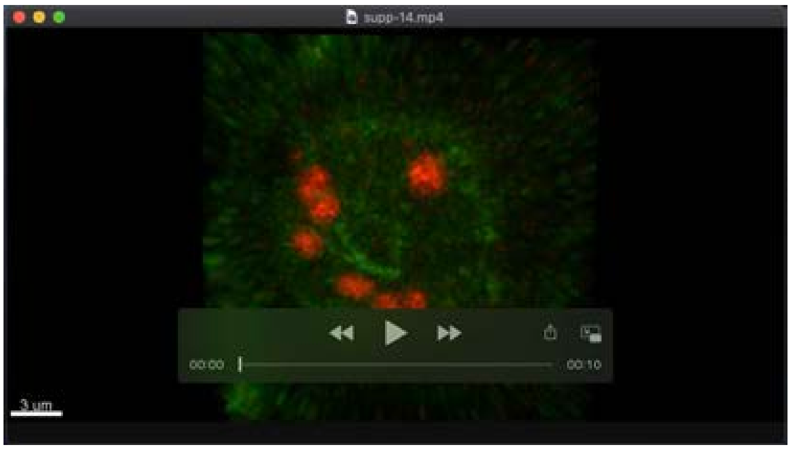

Movie 13

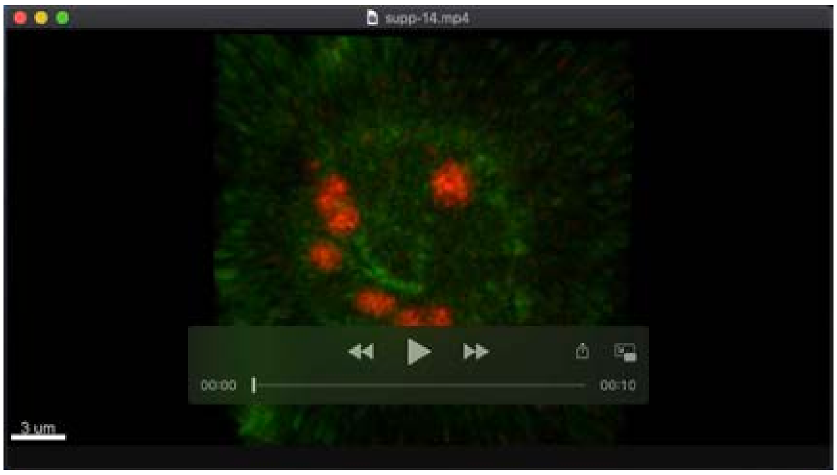

Movie 14

Movies 12, 13, and 14.

Three-dimensional rotation movies during meiosis I for live *zyg-9(RNAi)* oocytes expressing GFP::TBB-2 and mCherry::H2B.

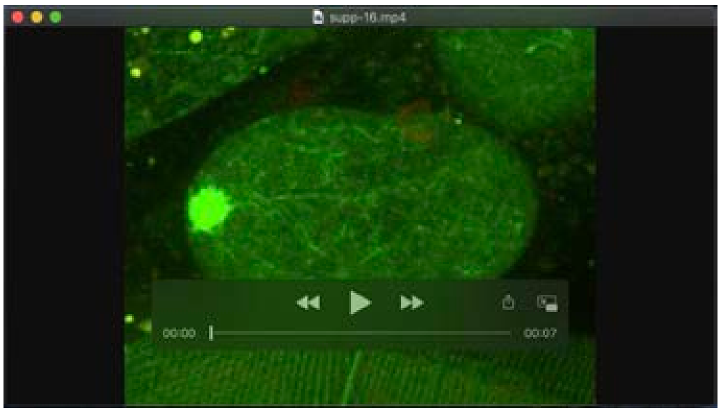

Movie 15.

Ex utero time-lapse spinning disk confocal maximum intensity z projection of 15 planes with 1 μm z-spacing movies during meiosis I for live control oocyte expressing GFP::TBB-2 and mCherry::H2B.

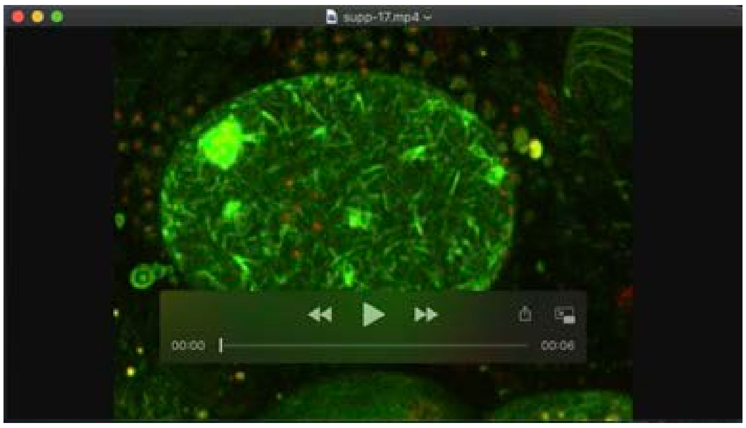

Movie 16.

Ex utero, time-lapse spinning disk confocal maximum intensity z-projection for 15 planes with 1 μm z-spacing movies during meiosis I of live *zyg-9(RNAi)* oocyte expressing GFP::TBB-2 and mCherry::H2B.

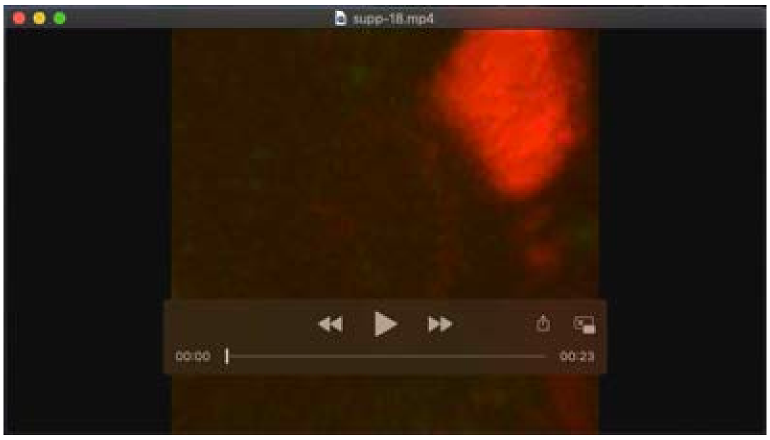

Movie 17

Time-lapse movie during meiosis I for live control oocyte expressing GFP::ASPM-1 and mCherry:: TBB-2.

Biology Open • Supplementary information

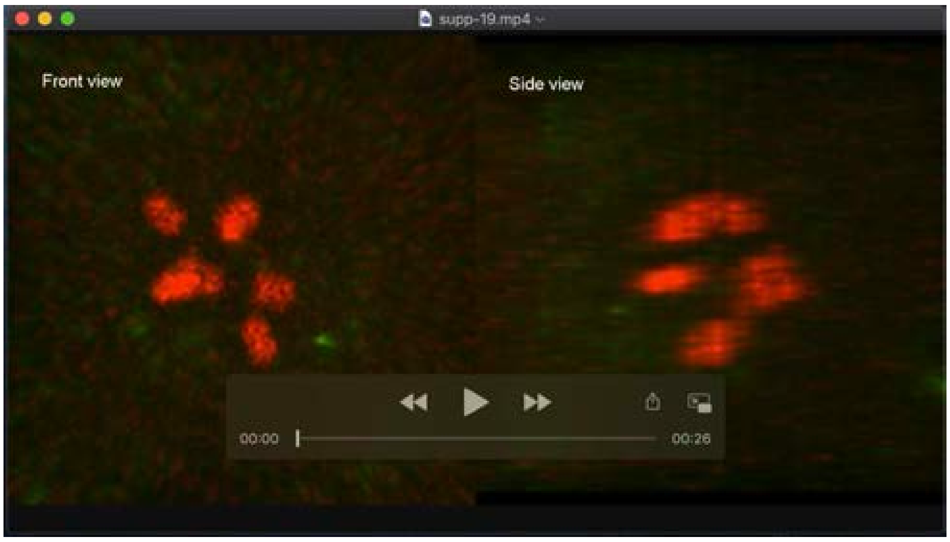

Movie 18

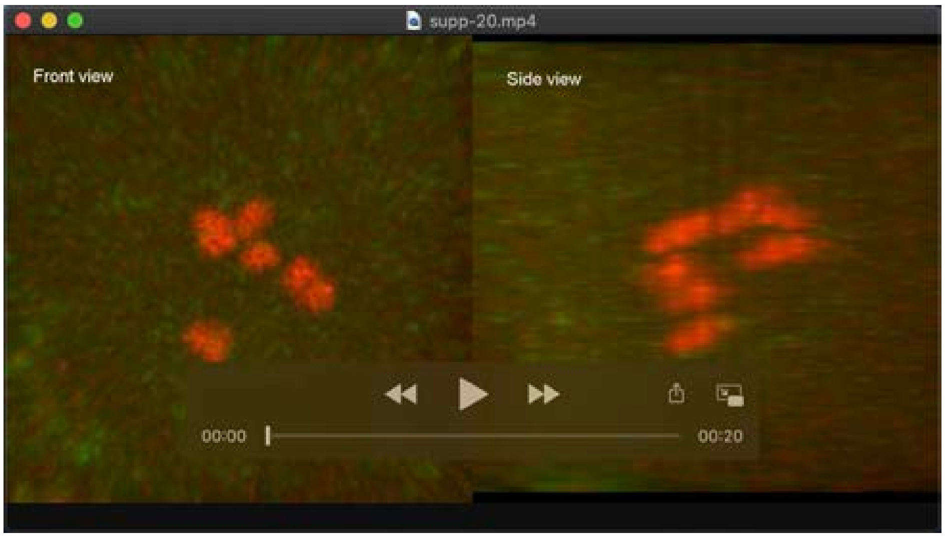

Movie 19

Movies 18 and 19.

Time-lapse movies during meiosis I for live *zyg-9(RNAi)* oocyte expressing GFP::ASPM-1 and mCherry::H2B.

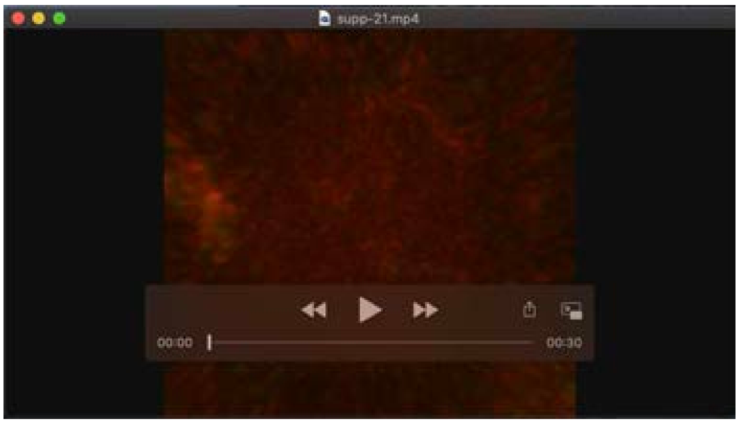

Movie 20

Time-lapse movies during meiosis I for live *zyg-9(RNAi)* oocyte expressing GFP::ASPM-1 and mCherry::TBB-2.

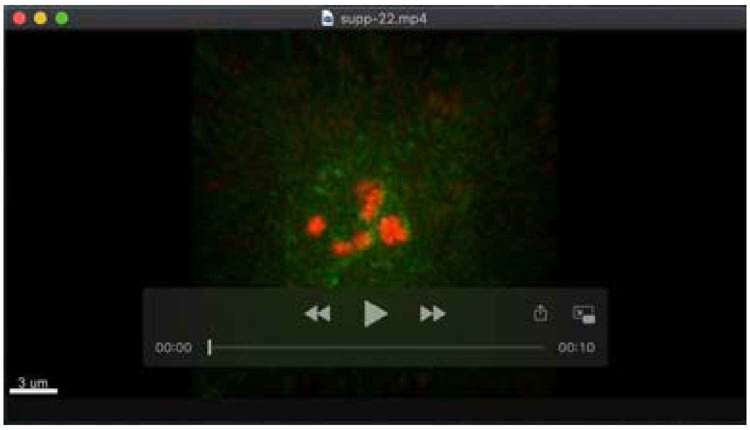

Movie 21

Three-dimensional rotation movie for live *tac-1(RNAi)* oocyte expressing GFP::TBB-2 and mCherry::H2B.

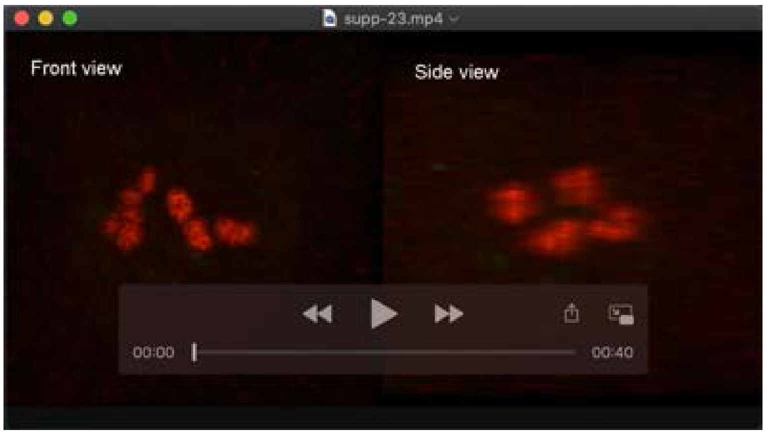

Movie 22

Time-lapse movies during meiosis I for live *tac-1(RNAi)* oocyte expressing GFP::ASPM-1 and mCherry::H2B.

Biology Open • Supplementary information

Table S1

Strain name	Genotype
N2	(ancestral)
EU2876	*aspm-1(or1935[gfp::aspm-1]) I; itIs37[pie-1p::mCherry::H2B::pie-1 3'UTR + unc-119(+)] IV*
EU2942	*ruIs57[pie-1p::gfp::tubulin + unc-119(+)]; itIs37[pie-1p::mCherry::H2B::pie-1 3'UTR + unc-119(+)] IV*
EU3006	*aspm-1(or1935[gfp::aspm-1]) I; tbb-2(tj29[mCherry::tbb-2]) III*
EU3091	*ijmSi49[pJD479; Pmex-5::gfp::lmn-1::3'tbb-2] I; tbb-2(tj29[mCherry::tbb-2]) III*
EU3115	*klp-15(ok1958) klp-16(or1952)/ tmC18[dpy-5(tmIs1236)] I; itIs37[pie-1p::mCherry::H2B::pie-1 3'UTR + unc-119(+)] IV; ruIs57[pie-1p::gfp::tubulin + unc-119(+)]*
EU3121	*tac-1(or1955[gfp::tac-1]) II; itIs37[pie-1p::mCherry::H2B::pie-1 3'UTR + unc-119(+)] IV (may contain unc-119(ed3) III)*
EU3169	*zyg-9(or1956[gfp::zyg-9]) II; itIs37[pie-1p::mCherry::H2B, unc-119(+)]IV (may contain unc-119(ed3) III)*
RB1593	*klp-15(ok1958)*
EU3201	*klp-15(ok1958) aspm-1(syb1260[gfp::aspm-1]) klp-16(or1952) /tmC18[dpy-5(tmIs1236)] I; itIs37[pie-1p::mCherry::H2B::pie-1 3'UTR + unc-119(+)] IV*

Biology Open • Supplementary information
